# Efficient potential-tuning strategy through p-type doping for designing cathodes with ultrahigh energy density

**DOI:** 10.1093/nsr/nwaa174

**Published:** 2020-07-27

**Authors:** Zhiqiang Wang, Da Wang, Zheyi Zou, Tao Song, Dixing Ni, Zhenzhu Li, Xuecheng Shao, Wanjian Yin, Yanchao Wang, Wenwei Luo, Musheng Wu, Maxim Avdeev, Bo Xu, Siqi Shi, Chuying Ouyang, Liquan Chen

**Affiliations:** Department of Physics, Laboratory for Computational Materials Physics, Jiangxi Normal University, Nanchang 330022, China; State Key Laboratory of Advanced Special Steel, School of Materials Science and Engineering, Shanghai University, Shanghai 200444, China; State Key Laboratory of Advanced Special Steel, School of Materials Science and Engineering, Shanghai University, Shanghai 200444, China; State Key Laboratory of Advanced Special Steel, School of Materials Science and Engineering, Shanghai University, Shanghai 200444, China; State Key Laboratory of Advanced Special Steel, School of Materials Science and Engineering, Shanghai University, Shanghai 200444, China; Department of Physics, Laboratory for Computational Materials Physics, Jiangxi Normal University, Nanchang 330022, China; Soochow Institute for Energy and Materials Innovations (SIEMIS), College of Physics, Optoelectronics and Energy & Collaborative Innovation Center of Suzhou Nano Science and Technology, Soochow University, Suzhou 215006, China; State Key Lab of Superhard Materials, College of Physics, Jilin University, Changchun 130012, China; Soochow Institute for Energy and Materials Innovations (SIEMIS), College of Physics, Optoelectronics and Energy & Collaborative Innovation Center of Suzhou Nano Science and Technology, Soochow University, Suzhou 215006, China; State Key Lab of Superhard Materials, College of Physics, Jilin University, Changchun 130012, China; Department of Physics, Laboratory for Computational Materials Physics, Jiangxi Normal University, Nanchang 330022, China; Department of Physics, Laboratory for Computational Materials Physics, Jiangxi Normal University, Nanchang 330022, China; Australian Nuclear Science and Technology Organisation, Kirrawee DC, NSW 2232, Australia; School of Chemistry, University of Sydney, Sydney 2006, Australia; Department of Physics, Laboratory for Computational Materials Physics, Jiangxi Normal University, Nanchang 330022, China; State Key Laboratory of Advanced Special Steel, School of Materials Science and Engineering, Shanghai University, Shanghai 200444, China; Materials Genome Institute, Shanghai University, Shanghai 200444, China; Department of Physics, Laboratory for Computational Materials Physics, Jiangxi Normal University, Nanchang 330022, China; Beijing National Laboratory for Condensed Matter Physics, Institute of Physics, Chinese Academy of Sciences, Beijing 100190, China

**Keywords:** p-type doping strategy, electrochemical potential tuning, Li(Na)BCF_2_/B_2_C_2_F_2_ cathodes, ultrahigh energy densities

## Abstract

Designing new cathodes with high capacity and moderate potential is the key to breaking the energy density ceiling imposed by current intercalation chemistry on rechargeable batteries. The carbonaceous materials provide high capacities but their low potentials limit their application to anodes. Here, we show that Fermi level tuning by p-type doping can be an effective way of dramatically raising electrode potential. We demonstrate that Li(Na)BCF_2_/Li(Na)B_2_C_2_F_2_ exhibit such change in Fermi level, enabling them to accommodate Li^+^(Na^+^) with capacities of 290–400 (250–320) mAh g^−1^ at potentials of 3.4–3.7 (2.7–2.9) V, delivering ultrahigh energy densities of 1000–1500 Wh kg^−1^. This work presents a new strategy in tuning electrode potential through electronic band structure engineering.

## Teaser

Teaser text: Band structure engineering is used to design a new family of transition-metal-free Li(Na)BCF^2^/B^2^C^2^F^2^ cathodes for rechargeable batteries that achieve a record-breaking high energy density.

## INTRODUCTION

**Figure 1.**
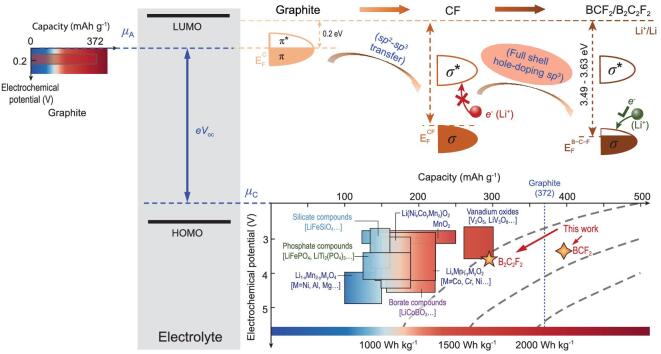
Schematic open-circuit voltage (V_oc_) of battery. The energy separation of the lowest-unoccupied-molecular-orbital (LUMO) and the highest-occupied-molecular-orbital (HOMO) is the electrolyte window. Electrochemical potential vs. capacity is presented for both graphite-anode and cathodes. The cathodes are commonly transition-metal (TM) compounds which have layered, spinel or olivine crystal structures.

The growing demand for batteries with exceedingly high energy density (ED) that can be used in electrical vehicles has inspired an active search—with regard to experiments and multi-scale calculations—for novel electrode materials [1–4]. The theoretical gravimetric energy density of a battery is defined as ${\rm{ED\ }} = \frac{{n{\rm{F}}{{\rm{E}}^0}}}{{\mathop \sum \nolimits {{\rm{M}}_i}}}\ $, where *n*, F, E^0^ and ΣM*_i_* refer to the electron transfer, Faraday constant, electromotive force and formula weights of active materials participating in the cell reaction, respectively [5]. To achieve higher energy density, a battery must be built of well-matched cathodes and anodes with: (i) a maximized amount of ions available for (*de*)intercalation, (ii) large Gibbs free energy change when charge carriers are transferred between electrodes, and (iii) a light weight [6]. Since anodes often offer higher Li-ion storage capacities than cathodes [7], there is an immense effort from both academia and industry to boost the capacities of current widely used transition-metal oxide (TMO) cathodes to above ∼300 mAh g^−1^ (Fig. 1). For a long time, it was considered that transition-metals (TMs) are the sole source of electrochemical activity in cathodes with the specific capacity being limited by the electrons that TMs can exchange [8–10]. However, recent studies demonstrated that oxygen in oxide cathodes may also participate in the redox reaction. Many Li-rich materials, such as Li_1+_*_x_*(Ni_1-_*_y_*_-_*_z_*Mn*_y_*Co*_z_*)_1-_*_x_*O_2_ [11,12], Li_1.2_Ni_0.2_Mn_0.6_O_2_ [5,13], Li_2_Ru_0.5_Sn_0.5_O_2_ [14] and Li_1.3_Mn_0.4_Nb_0.3_O_2_ [15], offer capacities up to 250–300 mAh g^−1^ through charge transfer via oxygen sites. However, the formation of highly reactive peroxide or superoxide species leads to potential safety issues, thus, the long-term practical application of these Li-rich oxides and viability of oxygen redox utilization is still under debate [16].

In light of the limited capacity of TMO cathodes, the advantages of light-weight carbonaceous materials as alternative electrodes are quite attractive. The variable orbital hybridization (*sp*, *sp^2^*, *sp^3^*) in those materials facilitates both structural stability and flexibility for Li^+^ storage [17]. With their operating potentials close to that of Li^+^/Li (0.2–1.5 V) and high theoretical capacity (e.g. 372 mAh g^−1^ for LiC_6_), they have served as ideal anode candidates for the past 25 years [18,19]. Other modified carbonaceous systems have also been proposed as high-capacity hosts for Li-ions, which can be classified into three types based on the carbon-hybridization nature (Table S1): 2D *sp^2^*, 3D *sp^2^* and 3D *sp^2^ + sp^3^* hybridization. Nevertheless, the Fermi levels of these modified carbon allotropes are all determined by the high-energy *sp^2^-p*_z_ orbitals (Fig. S1), resulting in the invariably low potentials. An interesting question remains though: can the potential of carbonaceous materials be tuned to a level comparable to those of currently used TMO cathodes?

Fluorides possess higher electrochemical potential than oxide analogs due to the strongly inductive effect of fluorine [20], which opens opportunities for modifying the potential of carbonaceous electrodes. Indeed, fluorinated-graphite (CF*_x_*) has been used as a cathode in Li primary batteries (Table S1) [21], whose potential reaches about 2.5 V [22]. Unfortunately, the lithiated fluorinated-graphite is rarely rechargeable, and its thermodynamic instability leads to spontaneous decomposition into graphite and LiF [23–25]. It is known that the chemical potential of lithium can be separated into the (electro)chemical potentials of electrons $\Delta {\mu _{{e^ - }}}$ and ions $\Delta {\mu _{L{i^ + }}}$. Here, $\Delta {\mu _{{e^ - }}}$ is determined by the Fermi level of electrode and plays a major role in the battery potential ( ${V_{{\rm{oc}}}} = \frac{{\Delta G}}{{zF}}{\rm{\ }} = \frac{{\mu _{Li}^A - \mu _{Li}^C}}{{zF}}\ = \frac{{\Delta {\mu _{{e^ - }}} + \Delta {\mu _{L{i^ + }}}}}{{zF}}\ $) [26]. Rational tuning of ion intercalation potentials of carbonaceous electrodes hence relies on fine control of their electronic structure [4,27,28]. So far, there is no report on improving the energy density of fluorinated-graphite cathodes for rechargeable batteries.

In this work, we use the graphite anode as a starting point to demonstrate the feasibility of the p-type doping strategy for shifting the Fermi level and substantially enhancing the potential of cathodes in an electrochemical cell. It is shown that the Fermi level drops upon p-type doping in the newly designed Li(Na)BCF_2_ and Li(Na)B_2_C_2_F_2_ cathodes. They have average Li^+^(Na^+^) deintercalation potentials up to 3.49 (2.78) V and 3.63 (2.85) V, and deliver theoretical capacities of 395.4 (319.6) mAh g^−1^ and 295.8 (251.2) mAh g^−1^, respectively. As a result, the gravimetric energy densities of Li(Na)BCF_2_ and Li(Na)B_2_C_2_F_2_ reach the record level of 1379.9 (888.5) and 1073.8 (715.9) Wh kg^−1^ for Li^+^(Na^+^) storage, respectively. This finding reveals an exciting path for the design of next-generation cathodes with ultrahigh energy density based on band structure engineering.

## RESULTS

The graphite anode exists in a relatively stable energy state with the Fermi level of −4.31 eV before Li^+^ intercalation (Fig. S2), which ultimately leads to its low potential (Fig. S1). Since the early 1970s there have been extensive efforts aimed at using fluorinated-graphite as cathodes for Li-ion batteries because of the strong electronegativity of fluorine [21]. The C-F *sp^3^* σ bond has a deep energy level, resulting in the lower Fermi level of CF (−6.97 eV) (Fig. S2). Considering that CF has a wide band gap (∼3.0 eV), the electrons introduced by lithiation can fill into low energy levels if holes are created, which may result in a high electrochemical potential (Fig. 1). Achieving this goal is complicated by the fact that holes need to be created under the following conditions: (i) p-doping (or hole-doping) should not fundamentally modify the favorable orbital hybridizations and, more importantly, (ii) the structure should be stable during the electrochemical process.

**Figure 2.**
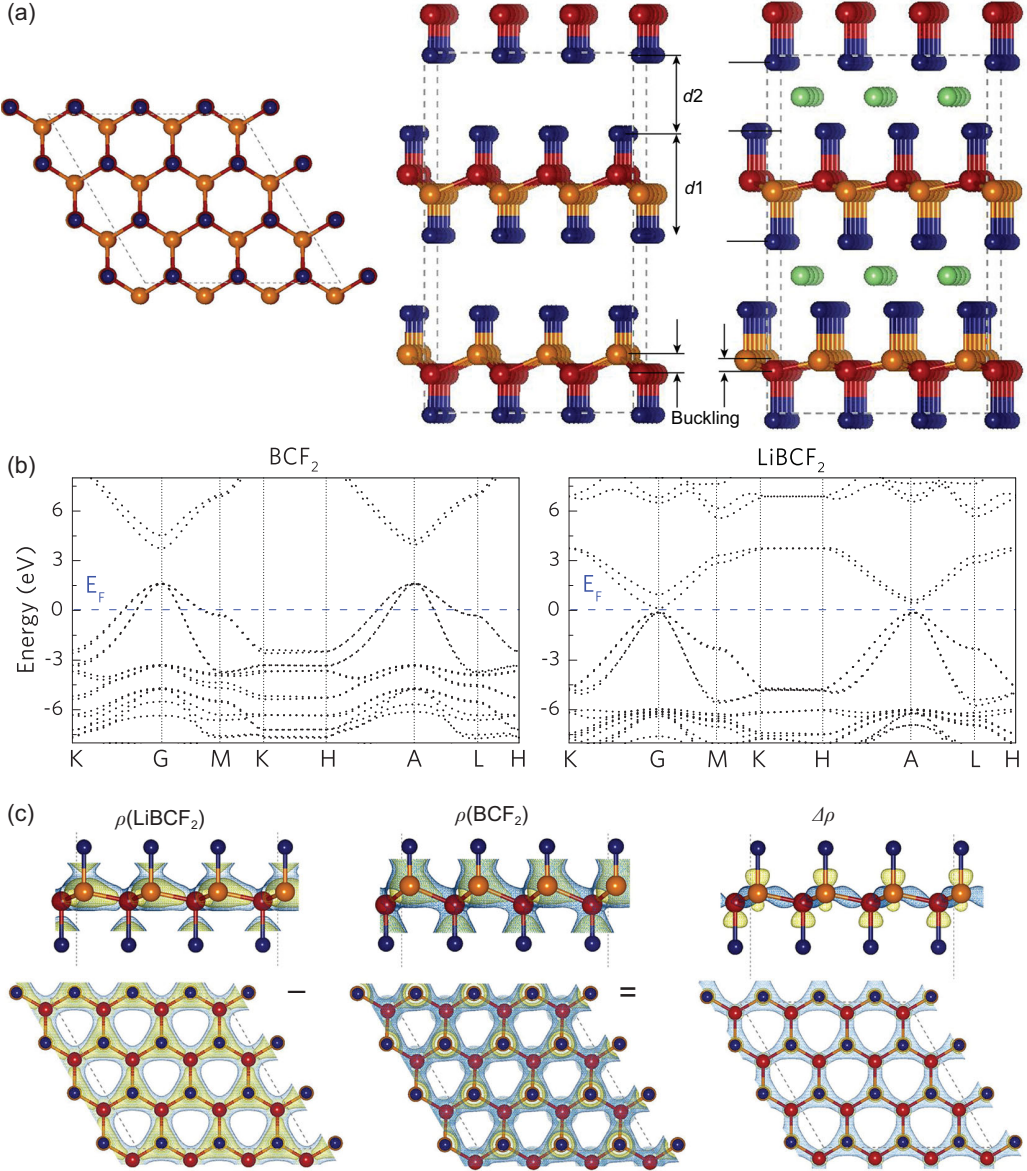
(a) Crystal structure of Li(Na)BCF_2_ cathodes. The yellow, red, blue and green spheres are C, B, F and Li(Na) atoms, respectively. The layer-thickness (*d*1) and layer-distance (*d*2) are also marked. (b) Band structures of BCF_2_ (left) and LiBCF_2_ (right). The Fermi level (E_F_) is set to zero. (c) The differential-charge-density (*Δρ*) between BCF_2_ and LiBCF_2_. The total charge density isosurfaces (0.032 e Å^−3^) of both BCF_2_ and LiBCF_2_ are given in yellow. The positive and negative isosurfaces (0.003 e Å^−3^) of *Δρ* are given in blue and yellow, respectively.

Being adjacent in the Periodic Table to C, B is an ideal dopant for creating holes in CF. By using an unbiased swarm-intelligence structure searching method, as implemented in the CALYPSO code [29], we identified stable structures in the Li(Na)-B-C-F system. The effectiveness of the CALYPSO code in finding layered materials has been validated by successfully reproducing known materials [30]. Many new materials predicted by CALYPSO have been also experimentally confirmed [30,31]. Following the procedure described in Section S3, we reproduced the known CF structures and identified new stable Li(Na)BCF_2_ with *P-*3*m*1 symmetry (Fig. 2 and Table 1).

**Table 1.** Lattice constants *a* and *c* (Å), volume V (Å^3^), buckling (Å), F-C-C bond-angle in CF and F-B-C bond-angle in B-alloying system (°), *d*1 (Å) and *d*2 (Å) in the Li(Na)BCF_2_/Li(Na)B_2_C_2_F_2_ cathodes. The data for CF are also given for comparison.

	BCF_2_	LiBCF_2_	NaBCF_2_	B_2_C_2_F_2_	LiB_2_C_2_F_2_	NaB_2_C_2_F_2_	CF
*a = b*	2.713	2.802	2.856	2.696	2.720	2.752	2.599
*c*	11.630	11.479	12.451	15.895	15.759	16.798	11.403
V	74.113	78.072	87.935	100.042	100.944	110.188	66.701
Buckling	0.665	0.338	0.338	0.728	0.532	0.528	0.488
Bond-angle	113.01	101.81	101.58	115.07	108.72	108.37	108.03
*d*1	3.364	3.539	3.527	5.502	5.627	5.615	3.249
*d*2	2.451	2.201	2.699	2.445	2.252	2.784	2.452

The dynamical and thermodynamic stability of Li(Na)BCF_2_ was further verified by conducting phonon calculations and molecular dynamic simulations, as well as by calculating the grand potential phase diagrams under different synthesis conditions (Figs S10–S14 and Tables S5–S8). For dynamical stability, in Fig. S11, no imaginary frequency has been found to show good dynamic stability in the phonon dispersion curves for the lithiated and delithiated states. Furthermore, we also performed the molecular dynamics (MD) simulation of lithiated and delithiated states at 500 K (Fig. S12). The results show that the structures of lithiated and delithiated states maintain well. For the lithiated state LiBCF_2_, we further increased the MD simulation temperature to 800 K. The results show that the structure also maintains well. The combination of results indicates that, like many available cathodes, Li(Na)BCF_2_ structures are metastable and can be synthesized by considering the entropic effect. The Li/Na-ion migration behavior in Li(Na)BCF_2_ cathodes under fully charged and discharged states were also investigated by the first-principles calculations (Figs S15 and S16, and Table S9). As shown in Fig. S16, the energy barriers of Li ion migration in the lithiated state LiBCF_2_ and delithiated state BCF_2_ are 0.67 and 0.57 eV, respectively. For Na ion migration, the corresponding energy barriers are 0.70 and 0.48 eV, respectively. The energy barriers of the Li/Na ion diffusion in BCF_2_ systems are close to those in common cathode materials that are used in battery applications. Thus, the Li/Na ion diffusion in Li(Na)BCF_2_ structure is acceptable for battery applications.

Next, we investigated the electronic structure of the hole-containing BCF_2_ system compared to that of the CF system. The F-C-B bond angle in BCF_2_ and the F-C-C bond angle in CF, as given in Table 1, indicate that the sp^3^-hybridized CF framework is maintained after B-alloying. Thus, the concept of ‘p-type doping’ is proposed to emphasize that both the structure and the orbital hybridization in the BCF_2_/B_2_C_2_F_2_ system are unchanged from that of the pristine CF system. More importantly, the C-*sp* and F-*p* orbitals in CF form a stable state filled with electrons following the 8-electron rule (Section S2). As a result, the Fermi level of BCF_2_ shifts to −8.36 eV (Fig. S3), much lower than that of CF (−6.97 eV). Moreover, the Fermi level passes through the valence bands of BCF_2_, which proves the success of our strategy of creating holes in valence bands (Fig. 2b). It is also confirmed that lowering the Fermi level in the B-doped BCF_2_ system is caused by the electron loss on the *sp^3^* σ orbitals, and the overall orbital hybridization pattern of the original CF system is not much affected (Section S2). Therefore, the B-alloyed CF structure is expected to have a substantially increased electrochemical potential. Also, the band structures of both Li(Na)BCF_2_ and Li(Na)B_2_C_2_F_2_ exhibit metallic characteristics (Figs S2 and S3), implying their good electrical conductivity as cathodes.

**Figure 3.**
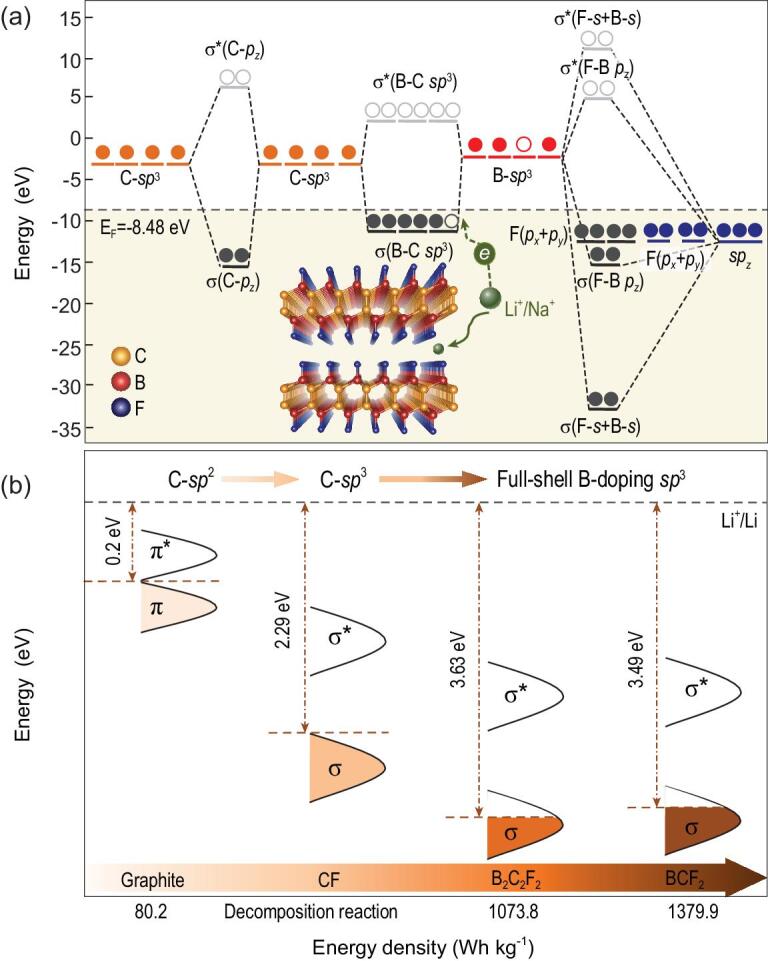
(a) Schematic of bonding modes between F, B and C orbitals in B_2_C_2_F_2_ system. All orbital energy levels are aligned with respect to the vacuum level. (b) Illustration of electronic structure tuning of carbonaceous electrode to address the high potential requirement for cathodes.

It is interesting to test if increasing the B/F ratio can further tune the electron chemical potential. On this basis, the B_2_C_2_F_2_ system, which was derived from another half-fluorinated graphite structure (C_2_F), was constructed. As shown in Table S2, the B-C hybridized orbitals are around the Fermi level in both BCF_2_ and B_2_C_2_F_2_ systems. Nevertheless, a longer B-C bond length (1.718 Å) was observed in B_2_C_2_F_2_ in comparison with that in BCF_2_ (1.702 Å). This is because half of the π bonds are passivated by F so the F-B interaction is relatively weak. Consequently, the Fermi level is slightly reduced from −8.36 eV in BCF_2_ to −8.48 eV in B_2_C_2_F_2_ (Fig. 3). The calculated grand potential phase diagram (Fig. S10) and phonon dispersion (Fig. S11) results also confirm that Li(Na)B_2_C_2_F_2_ can be experimentally prepared under certain pressure and temperature conditions.

We further investigate the effect of the Fermi level tuning on the ions-(*de*)intercalation potentials of both BCF_2_ and B_2_C_2_F_2_ systems. A new strategy based on group-subgroup analysis was proposed for a rigorous search over the large configurational phase space with unit-cells of up to 54 Li/Na sites for different intercalation stages of Li*_x_*(Na*_x_*)BCF_2_ and Li*_x_*(Na*_x_*)B_2_C_2_F_2_ (0 < *x* < 1, see details in Section S6). Through the process of subgroup projection and Wyckoff position splitting, 16 220 and 79 050 Li*_x_*/Na*_x_*BCF_2_ and Li*_x_*/Na*_x_*B_2_C_2_F_2_ configurations in different concentrations are obtained, respectively. Notably, the linear fitting residual of Ewald and density functional theory (DFT) energies shown in Fig. S19 implies that it makes no sense to compare the trend between DFT and Ewald energies. However, considering that the electrostatic energies should not be changed for structures that are symmetrically related, it is feasible to judge the structural similarity by using the calculated Ewald energies to save the computational resources. As a result, after screening and eliminating the symmetry equivalent structures by using the Ewald electrostatic energy method (Section S6.3), total energies of 2347 and 1337 configurations were calculated for the Li*_x_*/Na*_x_*BCF_2_ and Li*_x_*/Na*_x_*B_2_C_2_F_2_ ground state hull, respectively.

**Figure 4.**
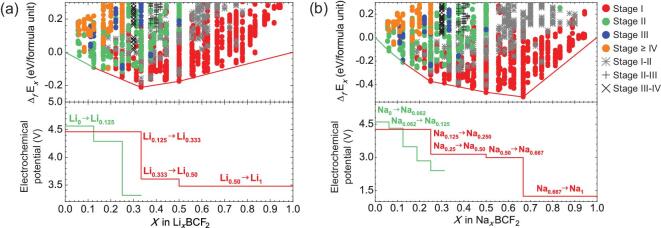
Formation energies (Δ*_f_*E*_x_*) and electrochemical potential of (a) Li*_x_*BCF_2_ and (b) Na*_x_*BCF_2_ as a function of Li^+^/Na^+^ concentration, respectively. Red and green solid lines indicate the constructed convex-hull of Stage I and Stage II phases, respectively.

Figure 4 and Tables S11–S14 show the stable configurations and corresponding formation energies (${\Delta _f}{{\rm{E}}_x}$ defined in Eq. S5) of Li*_x_*(Na*_x_*)BCF_2_ and Li*_x_*(Na*_x_*)B_2_C_2_F_2_. Some characteristics can be identified. First, the formation energies for all concentrations are negative, indicating the energetic stability of Li*_x_*(Na*_x_*)BCF_2_/B_2_C_2_F_2_. Second, different stages (defined as stage *x*, as discussed in Section S6.4) were found during both Li^+^ and Na^+^ intercalation into BCF_2_/B_2_C_2_F_2_ systems. This phenomenon has been reported in other 2D materials [32], and also has been experimentally confirmed in graphitic carbon [33]. Importantly, the average electrochemical potentials for Li^+^ (or Na^+^) intercalation in Li*_x_*(Na*_x_*)BCF_2_ and Li*_x_*(Na*_x_*)B_2_C_2_F_2_ were predicted to be 3.49 (2.78) V and 3.63 (2.85) V, respectively (Fig. 4 and Fig. S22). This confirms that the tuning of Fermi level indeed dramatically enhances the intercalation potentials of Li*_x_*(Na*_x_*)BCF_2_ and Li*_x_*(Na*_x_*)B_2_C_2_F_2_.

The stabilities of their deintercalated states Li*_x_*(Na*_x_*)BCF_2_/Li*_x_*(Na*_x_*)B_2_C_2_F_2_ (0 ≤ *x* ≤ 1) have been evaluated by calculating the formation energy of F vacancy (V_F_) (Section S6.5). In the temperature range of 200 to 500 K (a much wider range than the battery operating condition), where possible phase-transitions are solid-state reactions involving the absorption or loss of fluorine (μ(F) = 0 eV for Eq. S7), Li*_x_*(Na*_x_*)BCF_2_ and Li*_x_*(Na*_x_*)B_2_C_2_F_2_ cathodes remain stable [ΔH(V_F_) > 0] at all Li/Na concentrations (Fig. S25). Moreover, the compatibility of Li*_x_*(Na*_x_*)BCF_2_/Li*_x_*(Na*_x_*)B_2_C_2_F_2_ cathodes with the commonly used electrolyte is investigated. Here we take the classical 1 M LiPF_6_ in EC: DEC (1:1) electrolyte as an example. Its electrochemical window is reported to be larger than 4.5 V [4]. This is much higher than the Li^+^(Na^+^)-intercalation potentials of Li(Na)BCF_2_ and Li(Na)B_2_C_2_F_2_ cathodes (2.7–3.7 V) designed in our work, suggesting good compatibility between these cathodes and electrolyte.

**Figure 5.**
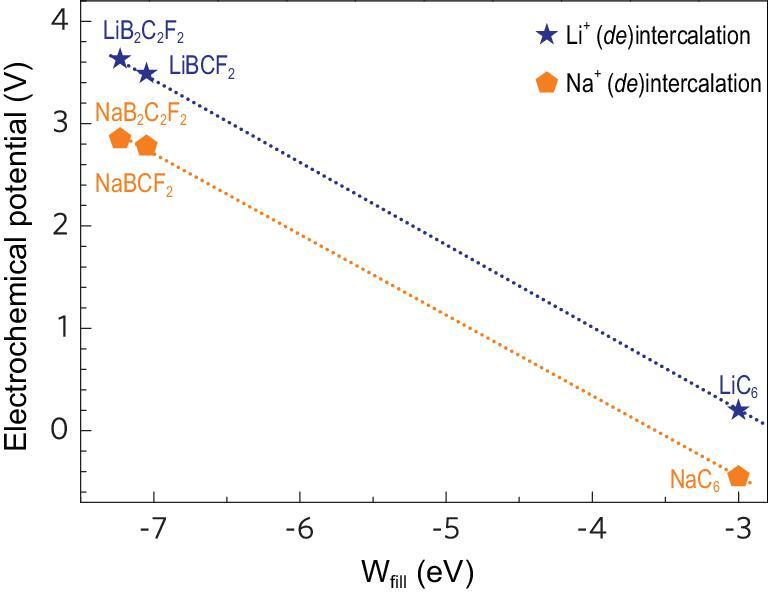
Electrochemical potentials as a function of W_fill_ for Li(Na)BCF_2_/Li(Na)B_2_C_2_F_2_ cathodes. Blue and yellow symbols represent Li^+^ and Na^+^ intercalation potentials, respectively. The dashed lines illustrate the dramatic increase of potentials of Li(Na)BCF_2_/Li(Na)B_2_C_2_F_2_ cathodes compared with that of graphite anode.

Considering that the intercalation process is mainly determined by charge transfer from Li/Na to BCF_2_ and B_2_C_2_F_2_ (Fig. S5), the energy gained in this transfer can serve as a descriptor to quantitatively illustrate the effect of p-type doping on the Li^+^/Na^+^ intercalation potentials. Also, the electrode potentials μ_A_ and μ_C_ constrain the open circuit voltage V_oc_ of a battery cell to $e{V_{oc}} = {\mu _A}\ - {\mu _C}$, where the energy of a given μ_A_ or μ_C_ corresponds to the Fermi energy in an itinerant-electron band, as is the case for Li metal, or the energy of a redox couple of the covalently bonded B-C cation in Li_x_(Na_x_)BCF_2_/Li_x_(Na_x_)B_2_C_2_F_2_. Thus, here we used the state-filling model [34] to calculate the energy acquired when Li/Na electron is transferred into an empty state above the Fermi level of pristine BCF_2_/B_2_C_2_F_2_. This can be described as ${{\rm{W}}_{{\rm{fill}}}} = \int_{{{E_F}}}^{{E^{\prime}}}{{{\rm{ED(E)}}/{{\rm{N}}_{{\rm{Li}}}}{\rm{dE}}}}$, where E is the energy referenced to the vacuum level, $D( {\rm{E}} )$ is the density of states of BCF_2_/B_2_C_2_F_2_ and ${\rm{E^{\prime}}}$ is derived from $\int_{{{E_F}}}^{{E^{\prime}}}{{D({\rm{E}}){\rm{dE}} = 1}}$, which assumes that one Li/Na electron is transferred into BCF_2_/B_2_C_2_F_2_ per formula unit. Note that the amount of charge transferred may not be precisely 1, as was previously demonstrated for other layered electrodes [35,36]. The charge transfer in BCF_2_/B_2_C_2_F_2_ was quantified by Bader analysis and the results are shown in Table S3. The amount of charge transferred from Na and Li to the B-C covalent bonds in BCF_2_/B_2_C_2_F_2_ is ∼0.78 and ∼0.86 in all cases, very close to 1. The W_fill_ results shown in Fig. 5 indicate that the high Fermi level (−4.31 eV) caused by the C-C *sp*^2^ hybridization in the pristine graphite leads to high W_fill_ (−3.0 eV) and ultimately lower electrochemical potential for Li^+^/Na^+^ (*de*)intercalation (<0.2 V). In contrast, p-type doping effectively lowers the Fermi level of BCF_2_ and B_2_C_2_F_2_ to −8.36 eV and −8.48 eV, respectively, and thus enables the systems to have lower W_fill_ (−7.05 eV for BCF_2_ and −7.23 eV for B_2_C_2_F_2_) and higher potentials. The relationship between the Fermi level and electrochemical potential quantitatively confirms the tuning effect of Fermi level on the intercalation potentials of Li^+^/Na^+^ in BCF_2_/B_2_C_2_F_2_ systems.

## CONCLUSION

In summary, we demonstrate how the rational p-type doping of full shell bonding-orbital in a CF system allows us to drastically shift Fermi level of structure to a lower energy, thus dramatically raising the electrochemical potential of the mother material. Adopting this concept, new cathode candidates Li(Na)BCF_2_ and Li(Na)B_2_C_2_F_2_ with average Li^+^(Na^+^) deintercalation potentials of 3.49(2.78) V and 3.63(2.85) V, respectively, were successfully designed. Most importantly, the theoretical energy densities of these cathodes reach 1379.9(888.5) and 1073.8(715.9) Wh kg^−1^ for Li^+^(Na^+^)-storage, which represent the highest values compared with any commercial cathodes currently used in Li-ion (or Na-ion) batteries.

We further suggest that this full shell p-type doping (or hole-doping) strategy can be applied to other charge transfer-dominated ion-intercalation systems. Indeed, a wide variety of the existing rigid-band transition metal dichalcogenides (TMD) can be considered for band structure tuning, including MX_2_ (M = Mo, W, Nb, Ta; X = S, Se) (Table S4). For example, Mo has six valence electrons (5*s*^2^4*d*^4^), which bond with 12 electrons of two stoichiometric S atoms (2 × 3*s*^2^3*p*^4^) to form an 18-electron full-shell configuration (Fig. S6). Notably, Nb has one less electron than Mo_,_ thus Nb substituting on Mo site can be regarded as p-type dopant for MoS_2_, shifting the Fermi level down as expected (Fig. S7). Upon Li^+^ intercalation process, the electron of Li fills lower energy levels, resulting in a higher potential in NbS_2_ (2.90 V) than in MoS_2_ (0.90 V) (Table S4). Thus, the full-shell doping strategy works well for both *p* and *d* elements. The findings in this work are expected to motivate more researchers to directly evaluate the link between the electrochemical potential and the band structure tuning of electrodes.

## Supplementary Material

nwaa174_Supplemental_File
