# Supplementary material for: Efficient potential-tuning strategy through p-type doping for designing cathodes with ultrahigh energy density
Source: Natl Sci Rev. 2020 Jul 27;7(11):1768–75. doi: 10.1093/nsr/nwaa174 (PMC8288616; doi:10.1093/nsr/nwaa174)
Supplement: nwaa174_Supplemental_File [file nwaa174_supplemental_file.docx]

Supplementary Materials for

# Efficient potential-tuning strategy through p-type doping for designing cathodes with ultrahigh energy-density

Zhiqiang Wang#1, 2, Da Wang#2, Zheyi Zou2, Tao Song2, Dixing Ni1, Zhenzhu Li4, Xuecheng Shao5, Wanjian Yin4, Yanchao Wang5, Wenwei Luo1, Musheng Wu1, Maxim Avdeev6, 7, Bo Xu1, Siqi Shi2, 3, *, Chuying Ouyang1, *, Liquan Chen8

1Department of Physics, Laboratory for Computational Materials Physics, Jiangxi Normal University, Nanchang 330022, China

2State Key Laboratory of Advanced Special Steel, School of Materials Science and Engineering, Shanghai University, Shanghai 200444, China

3Materials Genome Institute, Shanghai University, Shanghai 200444, China

4Soochow Institute for Energy and Materials Innovations (SIEMIS), College of Physics, Optoelectronics and Energy & Collaborative Innovation Center of Suzhou Nano Science and Technology, Soochow University, Suzhou 215006, China

5State Key Lab of Superhard Materials, College of Physics, Jilin University, Changchun 130012, China

6Australian Nuclear Science and Technology Organisation, Locked Bag 2001, Kirrawee DC, NSW 2232, Australia

7School of Chemistry, University of Sydney, Sydney 2006, Australia

8Beijing National Laboratory for Condensed Matter Physics, Institute of Physics, Chinese Academy of Sciences, Beijing 100190, China

#These authors contributed equally to this work.

*Corresponding author. Email: sqshi@shu.edu.cn (Siqi Shi); cyouyang@jxnu.edu.cn (Chuying Ouyang)

[Content](#_Toc36925303)

[Methods of calculations 3](#_Toc36925304)

[Section S1: Three types of graphite allotropes based on c-hybridization nature 4](#_Toc36925305)

[Section S2: Full shell p-doping tuning strategy for fluorinated graphite 5](#_Toc36925306)

[Section S3: Selection of Li/Na-BCF2 and Li/Na-B2C2F2, by using calypso 13](#_Toc36925307)

[Section S4: Thermodynamic stability of Li/Na-B-C-F systems 13](#_Toc36925308)

[S4.1 Grand potential phase diagrams 13](#_Toc36925309)

[S4.2 Reaction Gibbs free energy calculations along optimized reaction routes. 18](#_Toc36925310)

[Section S5: Diffusion properties 22](#_Toc36925311)

[Section S6: Electrochemical potential platform obtained by the group-subgroup analysis method 24](#_Toc36925312)

[S6.1 Algberic group description and subgroups classification of Li/Na-B-C-F 25](#_Toc36925313)

[S6.2 Wyckoff splitting for each pair *G* > *H*. 27](#_Toc36925314)

[S6.3 Configurations screening 27](#_Toc36925315)

[S6.4 Formation energy calculations 30](#_Toc36925316)

[S6.5 Stability of Li*x*(Na*x*)-B-C-F cathodes upon Li(Na)-ions (de)intercalation 38](#_Toc36925317)

[References 40](#_Toc36925318)

### Methods of calculations

The density functional theory calculations were carried out using the Vienna *ab-initio* simulation package (VASP).([1](#_ENREF_1)) The core ion and valence electron interaction is described by the projector augmented wave (PAW) method.([2](#_ENREF_2)) The generalized gradient approximation (GGA) expressed by the PBE functional([3](#_ENREF_3)) and a 550 eV cutoff energy for the plane-wave basis set were used in all computations. The first Brillouin zone was sampled with a Monkhorst-Pack special *k*-point mesh,([4](#_ENREF_4)) the density of the *k*-mesh ensures that the interval of the grid is less than 0.03 Å-1. The lattice parameters and the atomic position are fully relaxed, and the final Hellmann-Feynman forces on all relaxed atoms are less than 0.01 eV Å-1. The van der Waals (vdW) interaction is included for B, C, and F atoms by the PBE+D2 method with the Grimme vdW correction.([5](#_ENREF_5)) As Li/Na atoms are fully ionized and thus vdW corrections are not considered for them.

The calculation of the density of state (DOS) is smeared by the Gaussian smearing method with a smearing width of *k*BT = 0.05 eV. The optimized Li/Na (and vacancies) migration pathways and the migration energy barriers are obtained with the nudged elastic band (NEB) method.([6](#_ENREF_6)) The phonon frequencies are calculated using the PHONOPY package([7](#_ENREF_7)) which uses the force constants as inputs obtained from density functional perturbation theory (DFPT)([8](#_ENREF_8)) as implemented in the VASP. In order to obtain reliable phonon frequencies and dispersion data, technically very high accuracy and a relatively large unit cell should be used. To save the computational expense, the phonon dispersion data are obtained using a single layer sheet. Bader charge is used to analyze the charge distribution and allocation.([9](#_ENREF_9)) The first-principles molecular dynamics (FPMD) simulations are carried out using the NVT ensemble, which is controlled with a Nosé-Hoover thermostat.([10](#_ENREF_10)) After the BCF2 system reaches its equilibrium state, the temperature keeps at ~500 K for 18 *ps* (9000 MD steps). After the LiBCF2 system reaches its equilibrium state, the temperature keeps at ~500 K for 20 *ps* (10000 MD steps) and then increases to ~800K for another 10 *ps* (5000 MD steps).

### Section S1: Three types of graphite allotropes based on c-hybridization nature


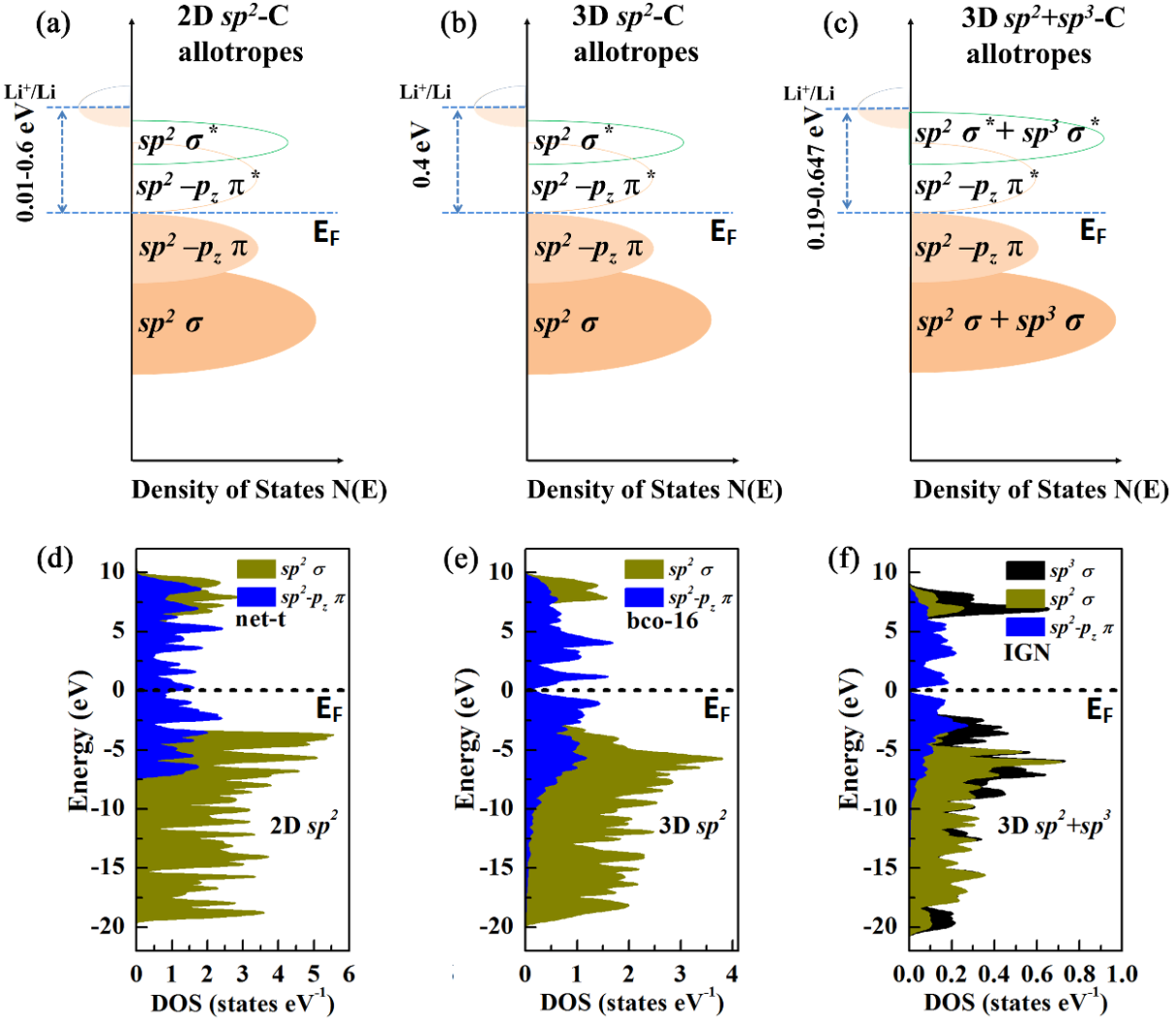


Figure S1. Representations of electronic structure in (a) 2D *sp2*-C allotropes, (b) 3D *sp2*-C allotropes and (c) 3D *sp2*+*sp3*-C allotropes, respectively. Schematic of their corresponding energy *vs*. density of states showing the relative positions of the Fermi energy (EF) in an itinerant electron band for Li insertion. (d), (e), (f) show the *sp2* σ, *sp2*-*pz* π and *sp3* σ partial density of states (PDOS) of three typic kind of carbon allotropes, 2D *sp*2-C, 3D *sp*2-C and 3D *sp*2+*sp*3-C, respectively.

Table S1. The electronic state, capacity (mAh g-1), structure and hybrid type, controlling orbital of Fermi level and electrochemical potential (V) of typical carbon allotropes electrodes for LIBs.

| Materials | Electronic State | Capacity (mAh g-1) | Structure and hybrid type | Controlling orbital of Fermi level | Electrochemical potential (V) |
| --- | --- | --- | --- | --- | --- |
| Graphene([11](#_ENREF_11)) | Semimetal | 214 | 2D *sp2* | *sp2*-*pz* | 0.11 |
| Penta-graphene([12](#_ENREF_12)) | Metal | 1489 | 2D *sp2* | *sp2*-*pz* | 0.24-0.6 |
| ψ-Graphene([13](#_ENREF_13)) | Metal | 372 | 2D *sp2* | *sp2*-*pz* | 0.01 |
| Net-w([14](#_ENREF_14)) | Metal | 1675 | 2D *sp2* | *sp2*-*pz* | 0.42 |
| Phagraphene([15](#_ENREF_15)) | Metal | 558 | 2D *sp2* | *sp2*-*pz* | 0.42 |
| Popgraphene([16](#_ENREF_16)) | Metal | 1487 | 2D *sp2* | *sp2*-*pz* | 0.19 |
| Net- t([17](#_ENREF_17)) | Metal | 558 | 2D *sp2* | *sp2*-*pz* | 0.04 |
| Biphenylene([18](#_ENREF_18)) | Metal | 623.72 | 2D *sp2* | *sp2*-*pz* | 0.4 |
| Graphdiyne([19](#_ENREF_19)) | Metal | 744 | 2D *sp2* | *sp2*-*pz* | 0.1-0.6 |
| bco-C16([20](#_ENREF_20)) | Metal | 558 | 3D *sp2* | *sp2*-*pz* | 0.23 |
| Hex-C18([21](#_ENREF_21)) | Metal | 496 | 3D *sp2*+*sp3* | *sp2*-*pz* | 0.47 |
| IGN-carbon([22](#_ENREF_22)) | Semi-metal | 298 | 3D *sp2*+*sp3* | *sp2*-*pz* | 0.41 |
| HZGM-42([23](#_ENREF_23)) | Semi-metal | 637.71 | 3D *sp2*+*sp3* | *sp2*-*pz* | 0.19 |
| CF | Semiconductor | Decomposition Reaction | 3D *sp3* | C-*sp3* (*px+py*) | 2.5-3.5 |

### Section S2: Full shell p-doping tuning strategy for fluorinated graphite

In the form of sharing electrons, the main group elemental compounds make the s and p orbitals filled with electrons in a stable state conform to the 8-electron rule. In our research, for CF, two σ(C-*px*+C-*py*) covalent bonds are formed between C and C to share four electrons, and two σ(F-*pz*+C-*pz*) share the remaining four electrons, therefore the CF system meets the 8-electron rule, as shown in Figure S2. A similar B-C-F bonding situation was observed in both BCF2 and B2C2F2 structure, where the σ(C-*pz*+F-*pz*) and σ(B-*pz*+F-*pz*) orbitals in C-F and B-F covalent bonds share two electrons, respectively, however, the electron loss caused by B-doping leads to the generation of hole (3 electrons occupation) in the σ(C-*px*+*py*/B-*px*+*py*) B-C bonds. This finally leads to the 7-electrons configuration achieved in BCF2 and B2C2F2 systems. Notably, this transition from full shell 8 to 7 electrons configuration (*sp3* in CF to p-doping *sp3* in BCF2/B2C2F2) did not change the crystal structures as well as the orbital hybridizations, except reducing the Fermi energy level of BCF2/B2C2F2 system (Figure S3).

Besides, it remains to clarify that whether the one less electron effect or the substituted B-atom itself plays more important role in the reduction of the Fermi level in BCF2 system. Thus, an artificial C2F2 system with one less electron removed from the C2F2 system (LE-C2F2), which has the same number of electrons with BCF2 system, was created by adding a background charge. In both BCF2 and LE-C2F2 systems (Figure S4), one hole on the fully-occupied C-*sp*3 (*s*+*px*+*py*)/C(or B)-*sp*3 (*s*+*px*+*py*) σ bonding orbital could be observed, where their band structures near Fermi level are almost the same, *e.g.*, the energy gap between C-*sp*3/C(or B)-*sp*3 σ bonding and antibonding state in LE-C2F2 and BCF2 is 3.0 eV and 2.89 eV, respectively, and also the bond angles determined by the B/C *sp3*-hybridization in these two systems are similar in the range of 113° to 115° (Table S2). On the basis of these findings, we can thus conclude that the decrease of Fermi level in BCF2 system is mainly caused by the electron loss on *sp3* σ bonding orbital without changing the orbital hybridization of the original CF system.

Notably, it is suggested that the p-doping of a full shell tuning strategy for band structure engineering could be generalized to other charge transfer-dominated ions intercalation systems. Notably, the important condition for its application is that it is best applied to the band rigidity systems that obey the full-shell 8-electron or 18-electron rule. One example is σ-binding of Li in LiFePO4 and LiMnPO4 systems, where both Fe and Mn have high spin electron arrangements, and the crystal structure in these two systems remains identical. However, the Li-2*s* electron localizing at the energetically lower *e*g spin-up orbital and energetically higher *t*2g spin-down orbital in Li*x*MnPO4 and Li*x*FePO4, respectively, create new σ-bonds in systems (Figure S9). This results in the unpredictable change of the average electrochemical potentials from 3.43 V of LiFePO4 to 4.13 V of LiMnPO4.


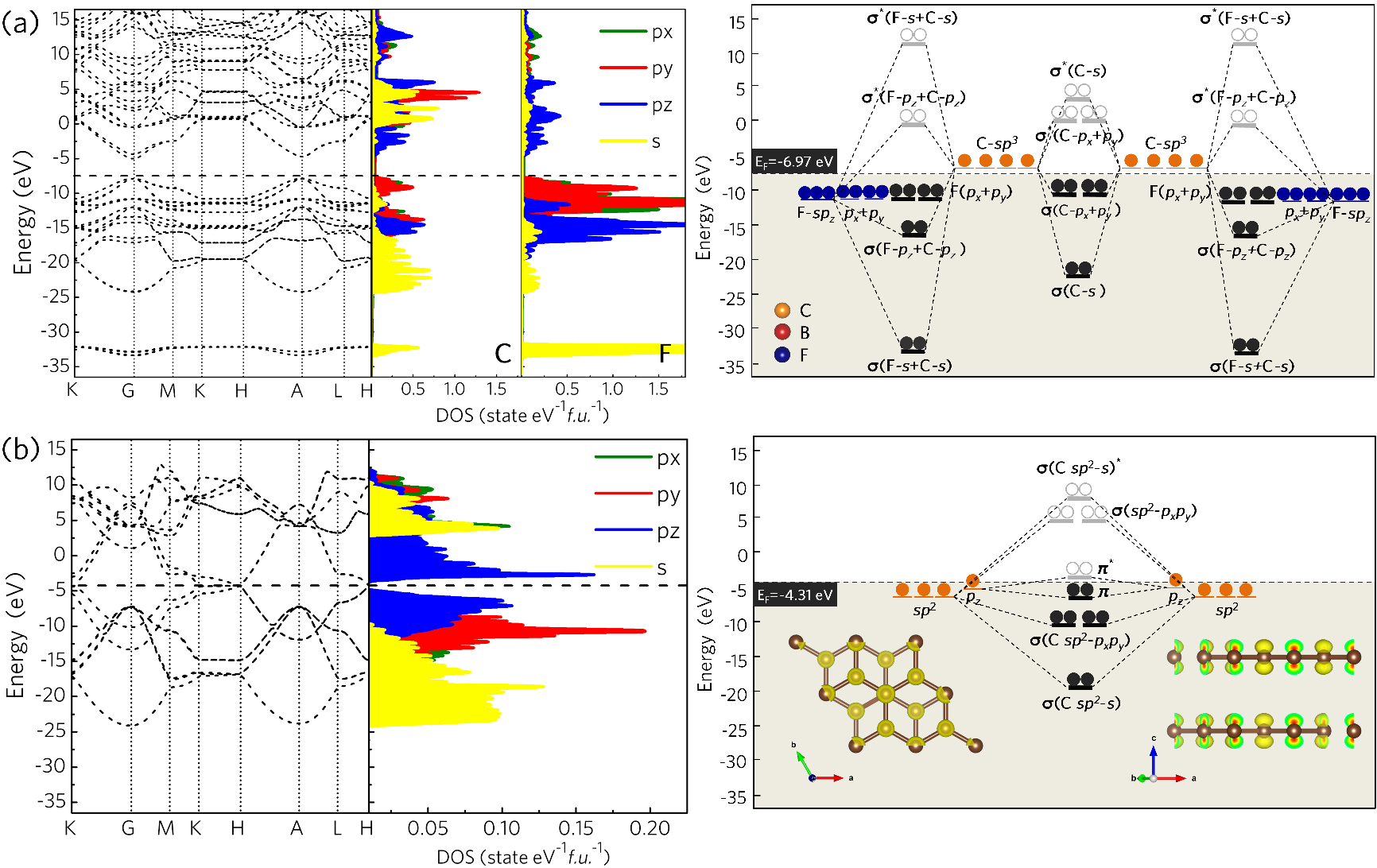


Figure S2. Schematic of the band structures and partial density of states (left), as well as the bonding modes between F and C orbitals (right) of (a) CF and (b) graphite, respectively. All of the orbital energy levels are aligned with respect to the vacuum level.


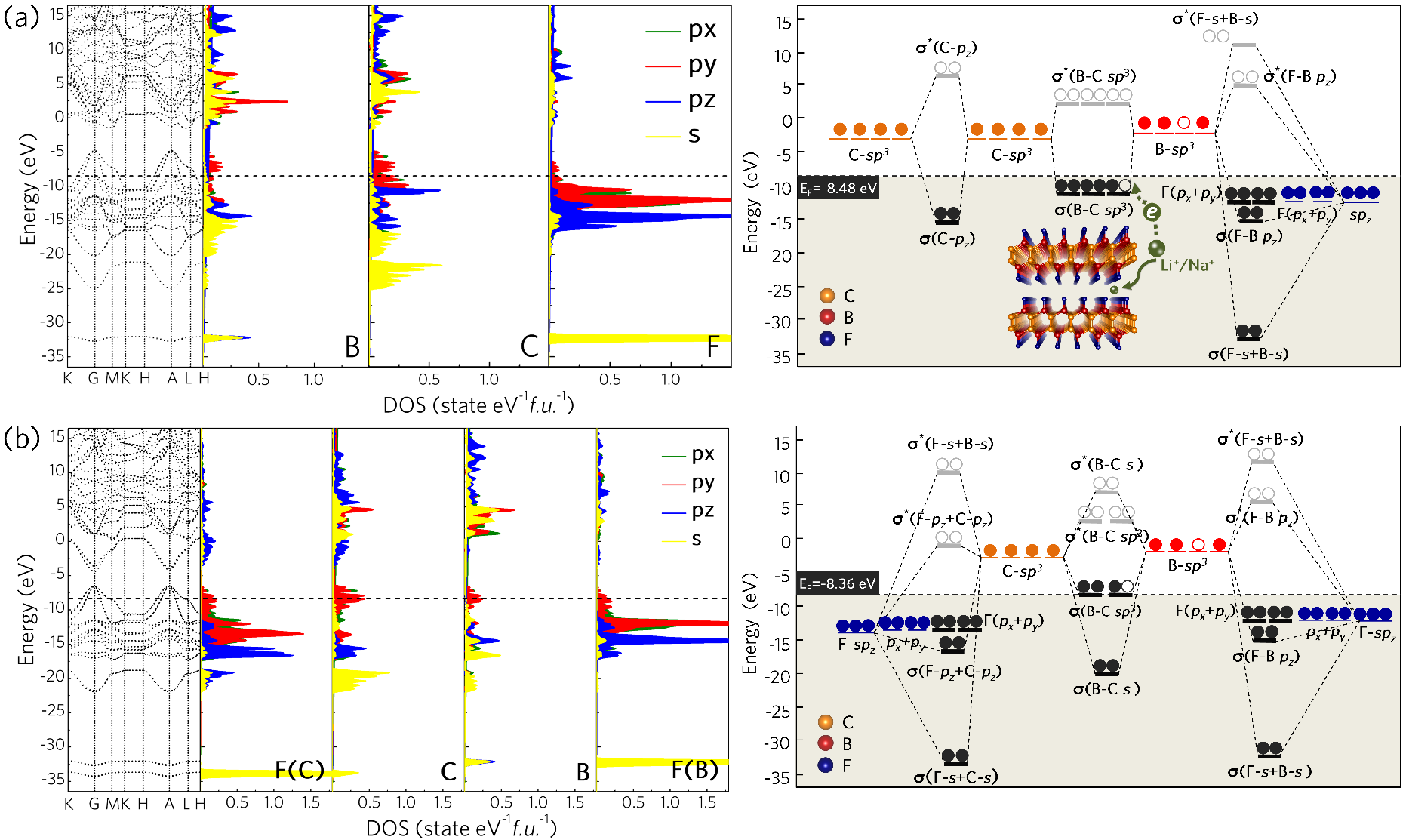


Figure S3. Schematic of the band structures and partial density of states (left), as well as the bonding modes between F, B and C orbitals (right) of (a) B2C2F2 and (b) BCF2, respectively. All of the orbital energy levels are aligned with respect to the vacuum level.


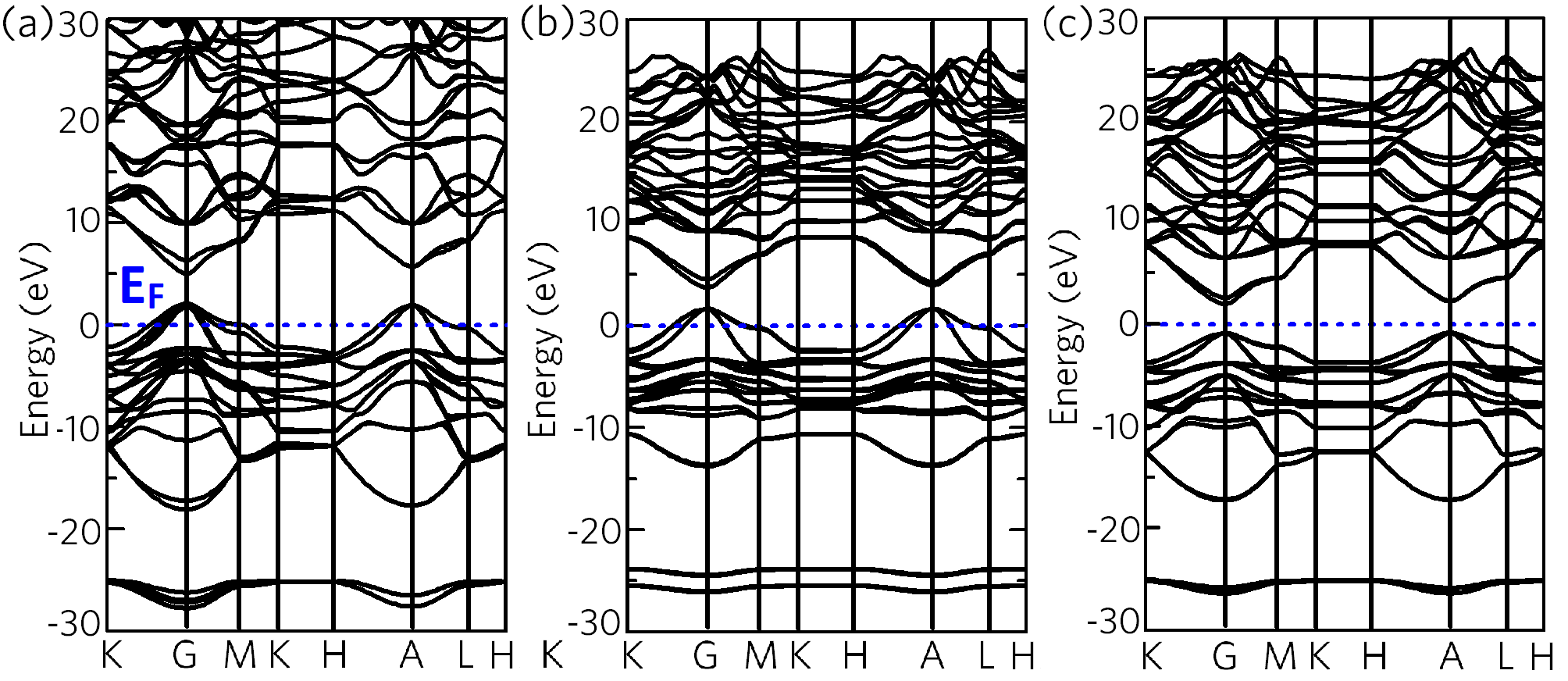


Figure S4. The band structures of (a) one less electron (LE) C2F2, (b) BCF2, and (c) CF, respectively. The Fermi level (EF) is set to be 0 eV.


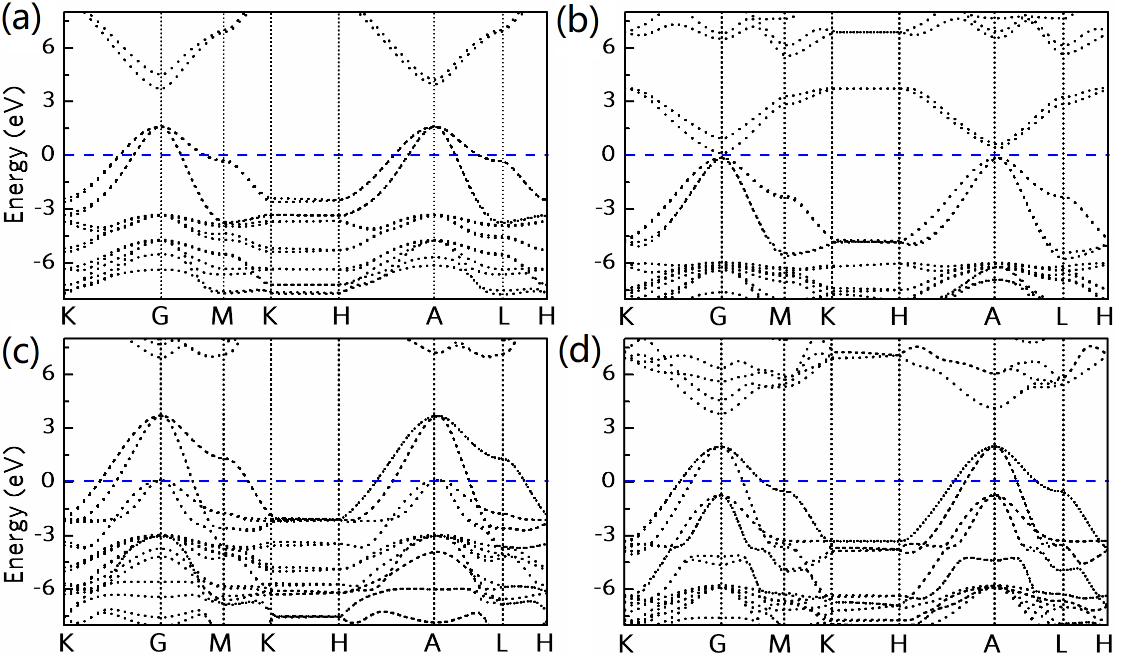


Figure S5. The band structures of (a) BCF2 and (b) LiBCF2, (c) B2C2F2 and (d) LiB2C2F2, respectively. The Fermi level (EF) is set to be 0 eV.


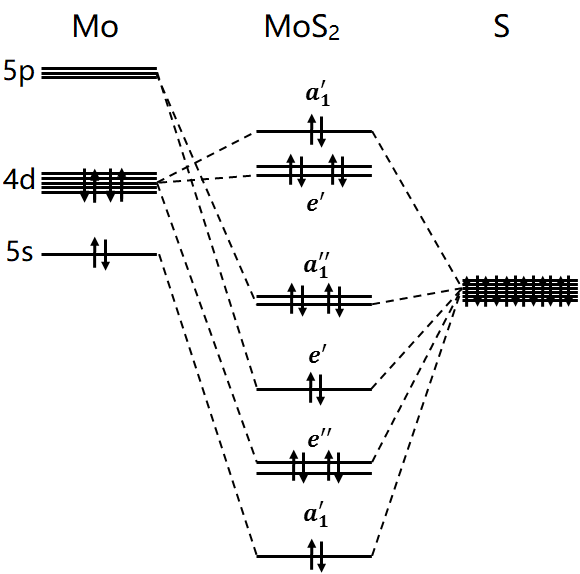


Figure S6. Schematic of the band alignment between Mo-4*d*/5*s*/5*p* and S-3*s*/3*p* in MoS2 system.


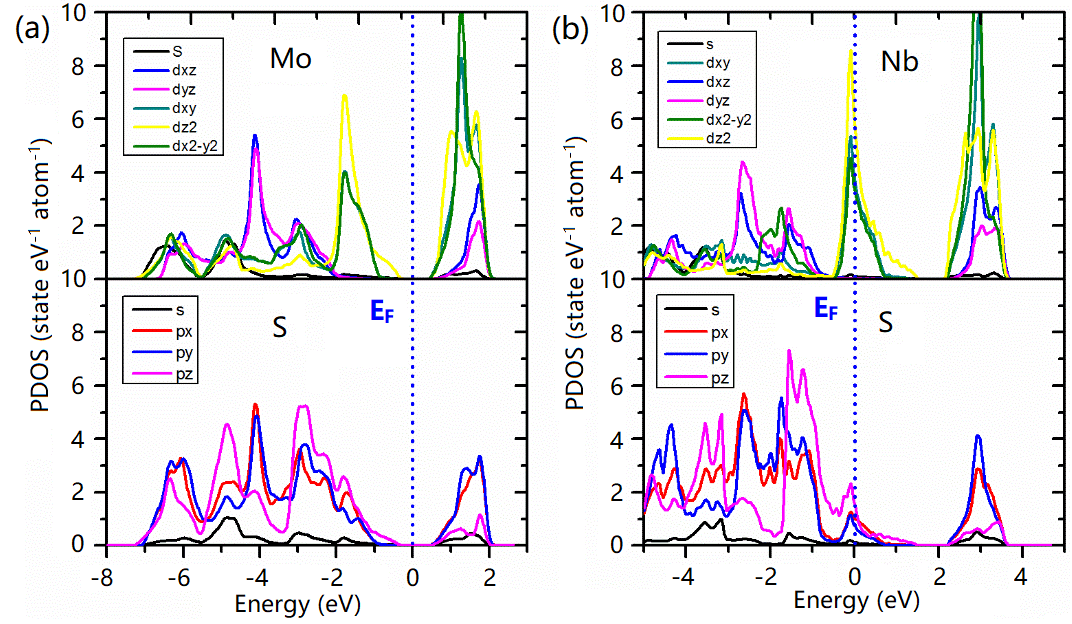


Figure S7. The partial density of states (PDOS) of (a) MoS2 and (b) NbS2, respectively. The Fermi level (EF) is set to be 0 eV.


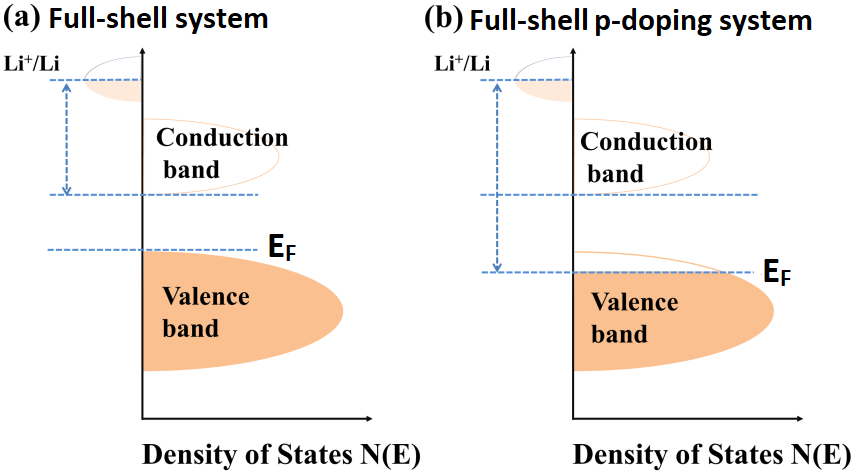


Figure S8. Schematic diagram of the p-doping effect on the shifting of fermi energy level in a full shell system. (a) and (b) represent the full-shell system and the full-shell p-doping system, respectively.


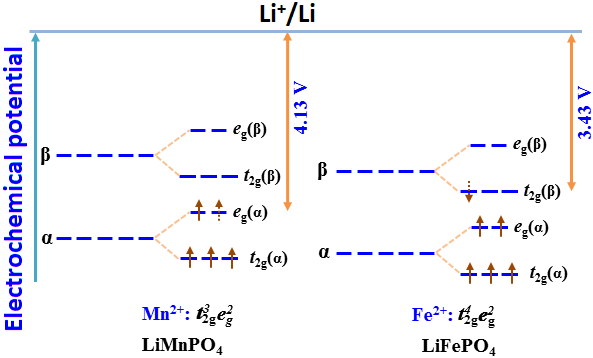


Figure S9. Crystal field splitting of the *M*2+ (*M* = Mn, Fe) cations in octahedral coordination. The Fe compound delivers a lower electrochemical potential *vs.* Li+/Li compared with the Mn compound, because the Fe2+/3+ redox energy shifts due to the pairing energy of the sixth electron in the *t*2*g* orbital.

Table S2. The lattice constants *a*(=*b*, in Å), the bond angle (in degree), the B-C (dB-C), B-F(dB-F), C-F (dC-F) and C-C (dC-C) bond lengths (in Å), for less-electron (LE) C2C2F2,C2C2F2,B2C2F2,LE-C2F2,BCF2 andC2F2, respectively.

|  | LE-C2C2F2 | B2C2F2 | C2C2F2 | LE-CCF2 | BCF2 | C2F2 |
| --- | --- | --- | --- | --- | --- | --- |
| *a=b* (Å) | 2.240 | 2.696 | 2.752 | 2.326 | 2.713 | 2.599 |
| Angle (°) | 116.26 | 115.07 | 108.37 | 114.7 | 113.01 | 108.03 |
| dB-F (Å) | -- | 1.333 | -- | -- | 1.343 | -- |
| dC-F (Å) | 1.225 | -- | 1.376 | 1.257 | 1.356 | 1.381 |
| dB/C-C (Å) | 1.442 | 1.718 | 1.560 | -- | 1.702 | -- |
| dC-C (Å) | 1.382 | 1.379 | 1.551 | 1.478 | -- | 1.578 |

Table S3. The Bader net charge of Li, Na, B, C and F atoms (in electrons) in different compounds. For comparison, the LiC2F2 is an artificial structure in which Li atoms are inserted into the lattice of the fluorinated graphite (C2F2) without relaxation of the atomic positions.

|  | Li/Na  (in electrons) | B  (in electrons) | C  (in electrons) | F  (in electrons) |
| --- | --- | --- | --- | --- |
| C2F2 | -- | -- | 0.58 | -0.58 |
| LiC2F2 | 0.86 | -- | 0.30 | -0.73 |
| BCF2 | -- | 1.84 | -0.51 | -0.66 |
| LiBCF2 | 0.86 | 1.87 | -1.23 | -0.75 |
| NaBCF2 | 0.78 | 1.80 | -1.16 | -0.71 |
| B2C2F2 | -- | 1.82 | -1.09 | -0.73 |
| LiB2C2F2 | 0.86 | 1.83 | -1.42 | -0.84 |
| NaB2C2F2 | 0.78 | 1.74 | -1.32 | -0.80 |

Table S4. The calculated electrochemical potentials of LiMPO4 (M = Fe or Mn) and MX2 (M = Mo, W, Nb, Ta; X = S, Se) electrodes.

| Electrodes | Electrochemical potential (V) | Electrodes | Electrochemical potential (V) |
| --- | --- | --- | --- |
| LiFePO4 | 3.43 | LiMnPO4 | 4.13 |
| MoS2 | 0.90 | NbS2 | 2.90 |
| WS2 | 0.70 | TaS2 | 2.70 |
| MoSe2 | 0.75 | NbSe2 | 2.40 |
| WSe2 | 0.50 | TaSe2 | 2.30 |

### Section S3: Selection of Li/Na-BCF2 and Li/Na-B2C2F2, by using calypso

In this work, we studied the structures and stabilities of selected compounds LiBCF2, LiB2C2F2, NaBCF2, and NaB2C2F2 using an accurate first-principles method while the particle swarm optimization algorithm implemented in the CALYPSO (Crystal structure AnaLYsis by Particle Swarm Optimization) method was used for the searching of stable LiBCF compounds.([24](#_ENREF_24), [25](#_ENREF_25)) The effectiveness and the efficiency of this crystal search method have been proven by many well-studied systems, including elements and binary and ternary compounds.([26](#_ENREF_26), [27](#_ENREF_27)) With the aid of this powerful tool, we obtained the most stable structures of the above selected compounds, as illustrated in Figure 2.

### Section S4: Thermodynamic stability of Li/Na-B-C-F systems

#### S4.1 Grand potential phase diagrams

Phase diagrams represent the thermodynamic phase equilibria of multicomponent systems and reveal useful insights into fundamental material aspects regarding the processing and reactions of materials. Ong *et al.*([28](#_ENREF_28), [29](#_ENREF_29)) outlined a thermodynamic methodology in which grand potential phase diagrams can be constructed from first principles calculated energies. The description of a closed four-component system at certain temperatures and chemical synthesis atmosphere requires three-dimensional space and can be represented in the form of a composition tetrahedron. However, given the large number of phases present in the Li/Na-B-C-F2 system, this representation is not one that is amenable to easy analysis. Therefore, the quaternary phase diagrams will be simplified as a plane ternary phase diagrams with a reasonable binary component. Considering only the entropy of gaseous phases, the phase diagram is constructed as a function of fluorination conditions, with the fluorine chemical potential, $\mu_{F_{2}}$, capturing both temperature and fluorine partial pressure dependence. Such phase diagrams represent phase equilibria in an isothermal, isobaric system that is open with respect to F2, which is representative of conditions during synthesis and operation of LiBCF2, LiB2C2F2, NaBCF2, and NaB2C2F2 cathodes.

Then, with the above information on phase relations garnered from the phase diagram, we study these reactions most relevant to phase stability for these given LiBCF2, LiB2C2F2, NaBCF2, and NaB2C2F2 phases, which rely on the convex-hull construction. The convex-hull construction effectively evaluates the stability of a given compound against any linear combination of compounds that have the same averaged composition, and is the common procedure to assess if a compound is stable versus the decomposition to other products.([30](#_ENREF_30)) First, we construct the convex hull with all phases existing in the Li/Na-B-C-F2 system. Then, we detect which phases constitute the equilibrium triangle in which the LiBCF2, LiB2C2F2, NaBCF2, and NaB2C2F2 composition lies. These reactions from these phases in the equilibrium triangle to form the LiBCF2, LiB2C2F2, NaBCF2, and NaB2C2F2 phases are defined as these reactions critical to phase stability. Indeed, they are these reactions that determine directly if the LiBCF2, LiB2C2F2, NaBCF2, and NaB2C2F2 phases are stable or not as they compare the LiBCF2, LiB2C2F2, NaBCF2, and NaB2C2F2 phases to the most competitive combination of phases.

In the Li/Na-B-C-F system, we are primarily comparing the relative stability of condensed phases, for which $P\Delta V$ is generally small and the $PV$ term may therefore be neglected and at 0 K, one would usually need to neglect all the relevant excitations with phase stability, such as vibrational, configurational, and electronic entropy. Therefore, the study of the optimal synthesis route will be simplified into the comparison of the ground state energy of the Li/Na-B-C-Fsystem with the decomposition products and the related competing phase.

To construct phase diagrams, we extract all structural prototypes and energies in the Li/Na-B-C-F system except LiBCF2, LiB2C2F2, NaBCF2, and NaB2C2F2 in the Materials Project Online Database (Table S5). All energies are presented as per formula unit (f.u.) formation energies, $E_{f}$, from the elements, Li, Na, B, C, and F2. The structures and energies of LiBCF2, LiB2C2F2, NaBCF2, and NaB2C2F2 studied in this paper are obtained by the first principle calculations, which are performed with Vienna *ab-initio* simulation package (VASP).([1](#_ENREF_1), [31](#_ENREF_31)) The obtained minimum reaction formation energies of LiBCF2 and NaBCF2 are 2.383 eV and 2.921 eV, respectively, which are thermodynamically unstable with respect to B13C2, C, LiF or NaF phases under special fluorine chemical potential ($\mu_{F_{2}}$), as presented in detail in Table S6. Thus, the stabilities of Li/Na-B-C-F phases under different pressures and temperatures were further investigated.

Table S5. Crystal structures and formation energies of phases in the Li/Na-B-C-F system.

| Phase | Crystal structure | Formation energy (eV/f.u.) | Phase | Crystal structure | Formation energy (eV/f.u.) |
| --- | --- | --- | --- | --- | --- |
| BC | $P6_{3}mc$ | 0.927 | Li5B4 | $Cm$ | -0.364 |
| BC5 | $I\bar{4}m2$ | 0.883 | Li3C | $Fm\bar{3}m$ | 0.321 |
| BC7 | $P\bar{4}m2$ | 0.938 | LiC | $Immm$ | -0.247 |
| B4C | $R\bar{3}m$ | -0.210 | LiC6 | $P6/mmm$ | -0.703 |
| B9C | $P1$ | 3.207 | LiC12 | $P6/mmm$ | -0.817 |
| B13C2 | $R\bar{3}m$ | -1.161 | LiBF4 | $P3_{1}21$ | -19.488 |
| BF2 | $P2_{1}/c$ | -7.755 | LiBC | $P6_{3}/mmc$ | -1.814 |
| BF3 | $P2_{1}/c$ | -12.250 | LiB6C | $Amm2$ | -2.564 |
| B17F27 | $P\bar{1}$ | -101.596 | LiB13C2 | $Imma$ | -3.153 |
| B5F6 | $I4_{1}/a$ | -22.276 | Li2B2C | $P\bar{4}m2$ | -0.300 |
| CF2 | $P\bar{1}$ | -4.678 | Na3B20 | $Cmmm$ | -2.612 |
| CF4 | $C2/c$ | -10.846 | NaB15 | $Imma$ | -1.586 |
| C2F | $R\bar{3}$ | -1.982 | NaBF4 | $Cmcm$ | -19.778 |
| C7F3 | $P\bar{1}$ | -5.524 | NaF | $Fm\bar{3}m$ | -6.373 |
| C10F3 | $Cc$ | -3.785 | Na2B29 | $Imm2$ | -1.219 |
| C11F7 | $C2/m$ | -12.034 | Na3C | $Fm\bar{3}m$ | 2.789 |
| C17F5 | $P2_{1}/m$ | -6.886 | NaB | $Fm\bar{3}m$ | 3.535 |
| C37F21 | $P1$ | -43.312 | NaC | $I4_{1}/acd$ | 0.160 |
| LiF | $Fm\bar{3}m$ | -6.714 | NaC64 | $P6_{3}/mmc$ | -0.265 |
| LiB | $P6_{3}/mmc$ | -0.631 | NaF3 | $I4/mmm$ | -6.514 |

Table S6. The most probable synthesis routes for LiBCF2, LiB2C2F2, NaBCF2 and NaB2C2F2.

| $\boldsymbol{\mu}_{\boldsymbol{F}_{\boldsymbol{2}}}$ (eV*)* | Reaction | Formation energy (eV/f.u.) |
| --- | --- | --- |
| -11.299 | 26LiF + 2B13C2 + 22C + 13F2 = 26LiBCF2 | 2.383 |
| -11.299 | 26LiF + 4B13C2 + 44C + 13F2 = 26LiB2C2F2 | 1.155 |
| -11.719 | 26NaF + 2B13C2 + 22C + 13F2 = 26NaBCF2 | 2.921 |
| -11.719 | 26NaF + 4B13C2 + 44C + 13F2 = 26NaB2C2F2 | 1.783 |


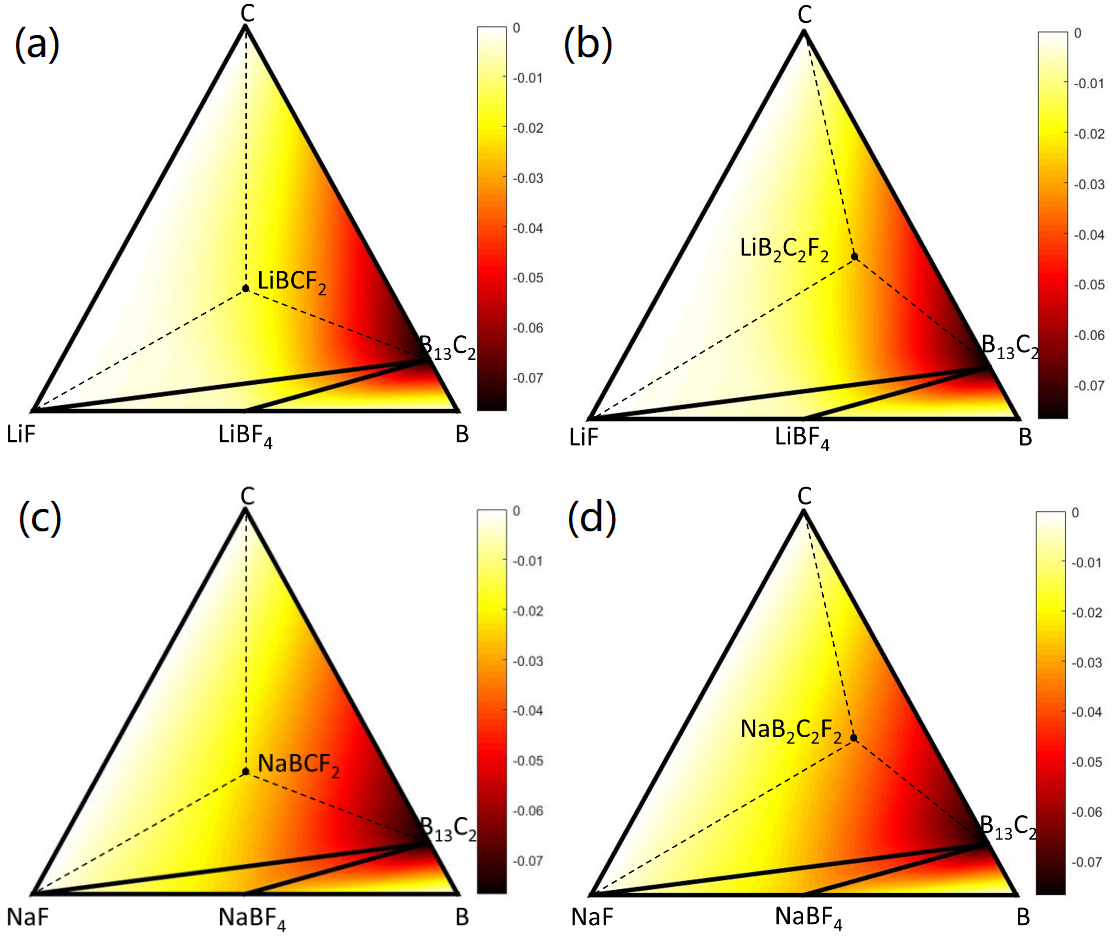


Figure S10. Grand potential phase diagrams for (a) LiBCF2, (b) LiB2C2F2, (c) NaBCF2 and (d) NaB2C2F2, under the F2 chemical potential ($\mu_{F_{2}}$) of -11.299 eV, -11.299 eV, -11.719 eV and -11.719 eV, respectively.


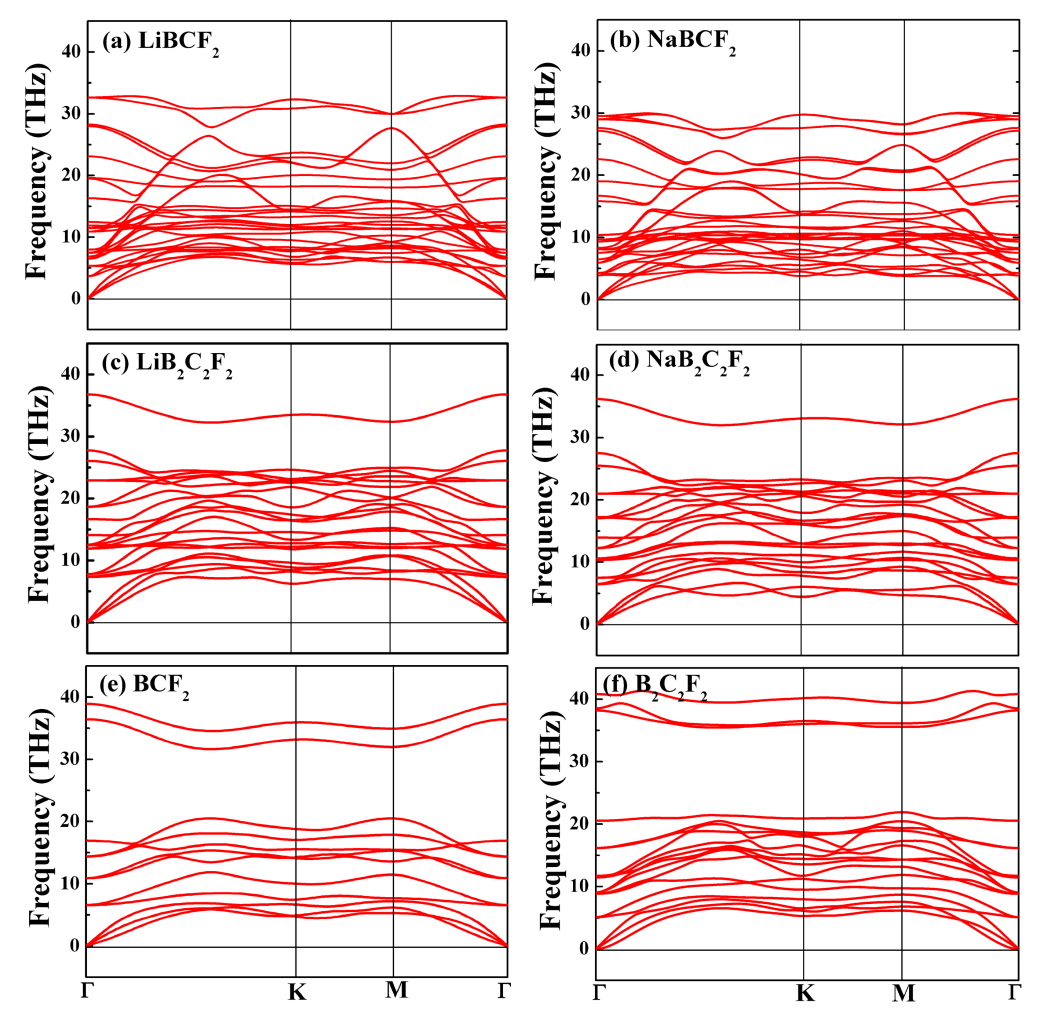


Figure S11. Phonon dispersion curves of Li/Na-BCF. (a) to (d) are for the discharged states LiBCF2, NaBCF2, LiB2C2F2, and NaB2C2F2, respectively. (e) and (f) are for the charged states BCF2 and B2C2F2, respectively.


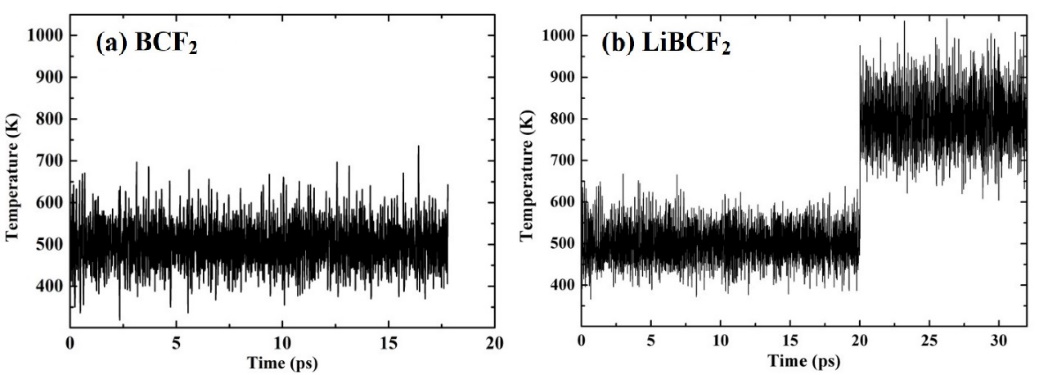


Figure S12. (a) Temperature variation during the MD simulation of the charged state (delithiated state) BCF2. The temperature keeps at ~500 K for 18 ps (9000 MD steps). (b) Temperature variation during the MD simulation of the discharged state (lithiated state) LiBCF2 cathode. The temperature keeps at ~500 K for 20 ps (10000 MD steps) and then increases to ~800K for another 10 ps (5000 MD steps).

#### S4.2 Reaction Gibbs free energy calculations along optimized reaction routes.

Along the optimized reaction route, the reaction Gibbs free energy has been calculated to evaluate thermodynamic stability for the Li(Na)*-*B-C-F systems. The total Gibbs free energy with respect to the temperature and pressure is expressed as followed:

$G(T,P)=G(T_{0},\Delta P)+G(\Delta T,P_{S})$ (Eq. S1)

where, the $G(T,P)$,$G(T_{0},\Delta P)$ and $G(\Delta T,P_{S})$are the total Gibbs free energy of the specific crystal structures with respect to the temperature and pressure, total energy with respect to the pressure and total energy dependent with the temperature. Thus, according to the optimized reaction functions, the reaction enthalpies $\Delta G(\Delta T,\Delta P)$ for XBCF (X=Li/Na) systems can be calculated as followed:

$\Delta G(\Delta T,\Delta P)=G_{LiBCF}(\Delta T,\Delta P)-G_{reagents}(\Delta T,\Delta P)$ (Eq. S2)

where the $G_{LiBCF}(\Delta T,\Delta P)$ and $G_{reagents}(\Delta T,\Delta P)$ are the total energy of XBCF (X=Li/Na) systems and that of the related reagents as a function of pressure and temperature, respectively. Thus, the entropic effects were introduced into the thermodynamic calculations for the relevant reagents and products. Here the lattice vibrational entropy at finite temperature is calculated from the phonon frequencies under the quasi-harmonic approximation. Furthermore, by introducing volume dependence of phonon frequencies, we calculate the reaction energies as a function of temperature and pressure. Because the F2 gas molecule is introduced as the external source and can be controlled by its [partial](D:/Youdao/Dict/8.4.0.0/resultui/html/index.html#/javascript:;) [pressure](D:/Youdao/Dict/8.4.0.0/resultui/html/index.html#/javascript:;)([29](#_ENREF_29)), it’s contribution to the reaction enthalpy have been adjusted in our thermodynamic calculations, as the results listed in Table S7. The results show that under the pressures up to 15GPa, the reaction enthalpy decrease with the increase of the temperature (Figure S13 and Table S8), indicating the preparation potential of Li/NaBCF2 under the specific pressure (~15 GPa) and temperature conditions.


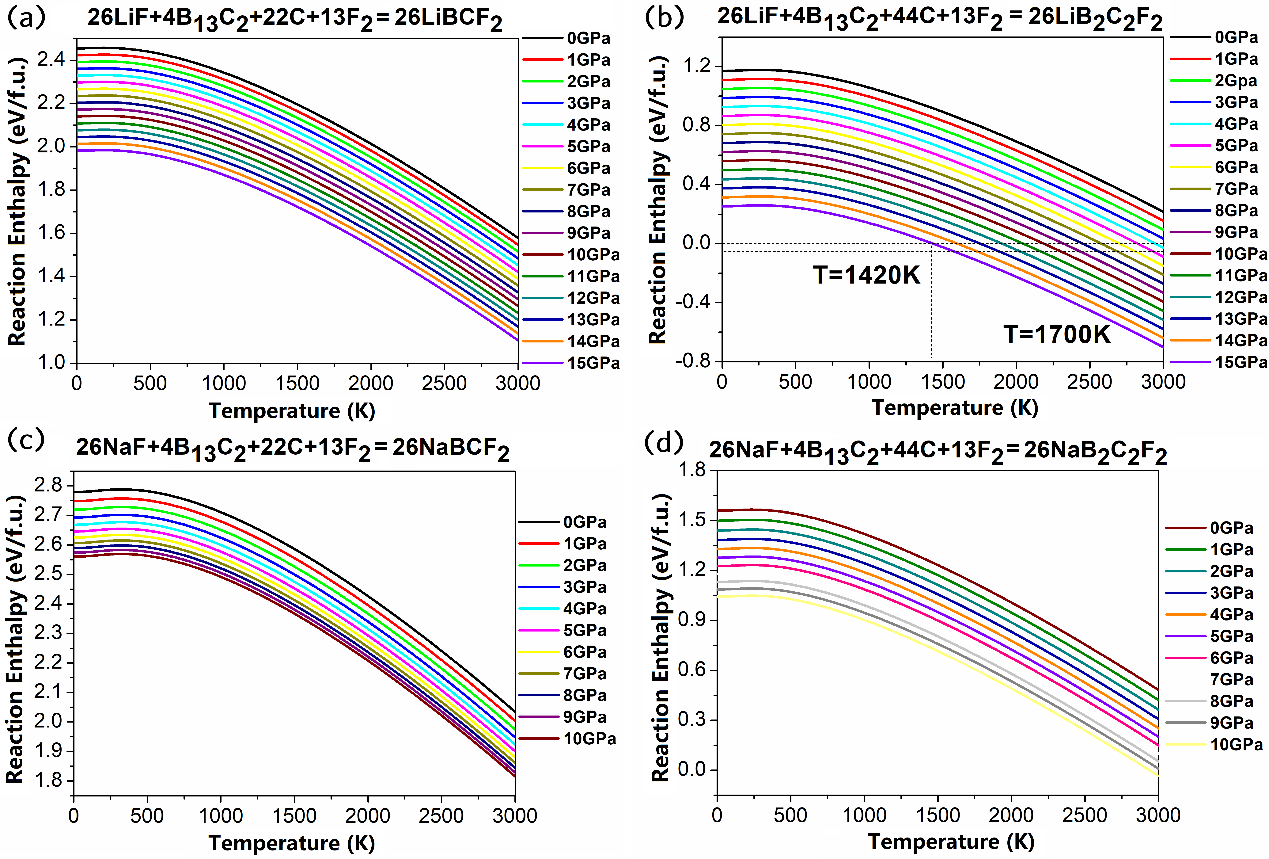


Figure S13. The reaction enthalpies of (a) LiBCF2, (b) LiB2C2F2, (c) NaBCF2 and (d) NaB2C2F2, respectively, as the function of temperature (0 K ≤ T ≤ 3000 K) and external pressure (0 GPa ≤ P ≤ 15 GPa).

Table S7. Pressure-dependent Gibbs free energy of $G_{P}(F_{2})$ for a F2 gas molecule as a function of pressure P (GPa).

| *P* (*GPa*) | *PV* (eV) | *GP(F2)* (eV) |
| --- | --- | --- |
| 0 | 0 | -11.298 |
| 1 | 0.120 | -11.178 |
| 2 | 0.240 | -11.058 |
| 3 | 0.361 | -10.938 |
| 4 | 0.481 | -10.817 |
| 5 | 0.601 | -10.697 |
| 6 | 0.721 | -10.577 |
| 7 | 0.841 | -10.457 |
| 8 | 0.962 | -10.337 |
| 9 | 1.082 | -10.216 |
| 10 | 1.202 | -10.096 |
| 11 | 1.322 | -9.976 |
| 12 | 1.442 | -9.856 |
| 13 | 1.563 | -9.736 |
| 14 | 1.683 | -9.615 |
| 15 | 1.803 | -9.495 |


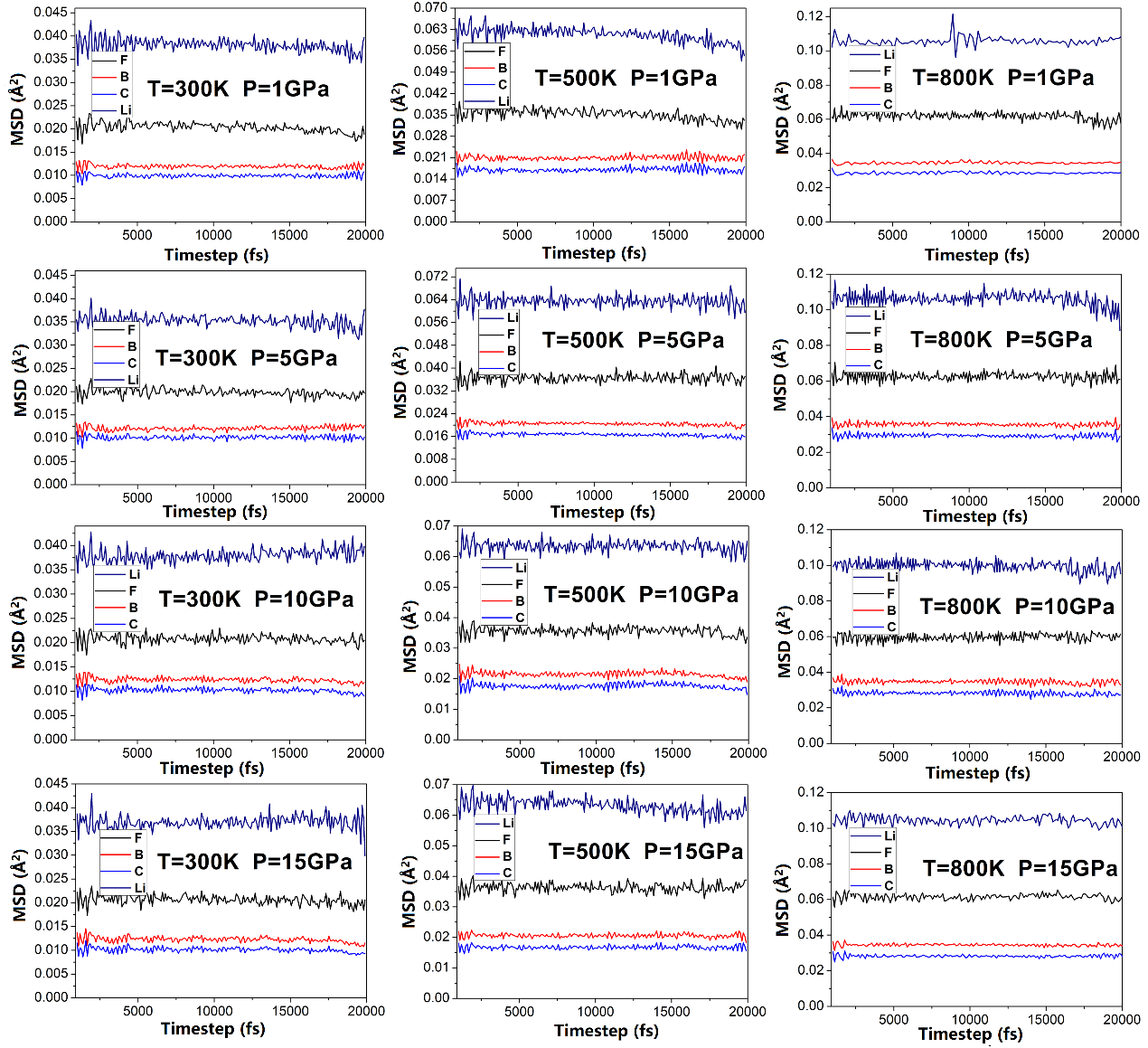


Figure S14. A first principles molecular dynamics simulations for the LiB2C2F2 system under the 300K, 500K, 800K with respect to the pressure of 1 GPa, 5 GPa, 10 GPa and 15 GPa, respectively.

Table S8. The pressure-dependent *PV* values and Gibbs free energies (*GP*) of C, B13C2, LiF, NaF, NaBCF2, NaB2C2F2, LiBCF2 and LiB2C2F2 as a function of pressure P (GPa), respectively.

| *P*  (*GPa*) | C | | B13C2 | | LiF | | NaF | | NaBCF2 | | NaB2C2F2 | | LiBCF2 | | LiB2C2F2 | |
| --- | --- | --- | --- | --- | --- | --- | --- | --- | --- | --- | --- | --- | --- | --- | --- | --- |
|  | *PV* (eV) | *GP* (eV) | *PV* (eV) | *GP*  (eV) | *PV* (eV) | *GP*  (eV) | *PV* (eV) | *GP* (eV) | *PV* (eV) | *GP*  (eV) | *PV* (eV) | *GP*  (eV) | *PV* (eV) | *GP*  (eV) | *PV* (eV) | *GP*  (eV) |
| 0 | 0.000 | -9.339 | 0.000 | -110.977 | 0.000 | -10.161 | 0.000 | -9.215 | 0.000 | -28.592 | 0.000 | -46.169 | 0.000 | -29.867 | 0.000 | -47.533 |
| 1 | 0.059 | -9.280 | 0.698 | -110.279 | 0.097 | -10.064 | 0.136 | -9.078 | 0.270 | -28.322 | 0.344 | -45.825 | 0.230 | -29.637 | 0.304 | -47.229 |
| 2 | 0.119 | -9.220 | 1.396 | -109.581 | 0.195 | -9.967 | 0.271 | -8.944 | 0.539 | -28.053 | 0.688 | -45.481 | 0.460 | -29.407 | 0.609 | -46.924 |
| 3 | 0.178 | -9.161 | 2.095 | -108.883 | 0.292 | -9.869 | 0.403 | -8.812 | 0.809 | -27.783 | 1.031 | -45.138 | 0.690 | -29.177 | 0.913 | -46.620 |
| 4 | 0.238 | -9.101 | 2.793 | -108.184 | 0.389 | -9.772 | 0.533 | -8.682 | 1.078 | -27.514 | 1.375 | -44.794 | 0.920 | -28.946 | 1.217 | -46.316 |
| 5 | 0.297 | -9.042 | 3.491 | -107.486 | 0.487 | -9.675 | 0.661 | -8.554 | 1.348 | -27.244 | 1.719 | -44.450 | 1.150 | -28.716 | 1.522 | -46.011 |
| 6 | 0.357 | -8.982 | 4.189 | -106.788 | 0.584 | -9.577 | 0.787 | -8.428 | 1.617 | -26.975 | 2.063 | -44.106 | 1.380 | -28.486 | 1.826 | -45.707 |
| 7 | 0.416 | -8.923 | 4.887 | -106.090 | 0.681 | -9.480 | 0.911 | -8.304 | 1.887 | -26.705 | 2.407 | -43.762 | 1.610 | -28.256 | 2.131 | -45.402 |
| 8 | 0.475 | -8.864 | 5.586 | -105.392 | 0.779 | -9.383 | 1.034 | -8.181 | 2.157 | -26.435 | 2.750 | -43.419 | 1.840 | -28.026 | 2.435 | -45.098 |
| 9 | 0.535 | -8.804 | 6.284 | -104.693 | 0.876 | -9.285 | 1.154 | -8.060 | 2.426 | -26.166 | 3.094 | -43.075 | 2.070 | -27.796 | 2.739 | -44.794 |
| 10 | 0.594 | -8.745 | 6.982 | -103.995 | 0.973 | -9.188 | 1.274 | -7.941 | 2.696 | -25.896 | 3.438 | -42.731 | 2.300 | -27.566 | 3.044 | -44.489 |

### Section S5: Diffusion properties

The migration barriers of ions in the systems are jointly determined by the Li(*s*)-host electron interaction caused by the crystallographic symmetry operations and the Coulomb repulsion energy of ions at different sites. Two different pathways were found for Li/Na-ions migration in both BCF2 and B2C2F2 systems, namely Path 1 (*Oh*→*D2h*→*Oh*) and Path 2 (*Oh*→*Td*→*Oh*), where Li/Na ion is six-coordinated with F atoms in low octahedral symmetry in initial and final state, labeled as *Oh* site and the transition state shows the octahedral site and tetrahedral site is labeled as *D2h* and *Td*, respectively (Figure S15). A summary of symmetry analysis is given in Table S9.

When Li/Na-ions occupies the initial octahedral site in both BCF2 and B2C2F2 compounds, the systems have *Oh* symmetry, which contains irreducible representations *a1g*, *a2g*, *eg*, *t1g*, *t2g*, *a1u*, *a2u*, *eu*, *t1u* and *t2u*. At the center of Brillouin zone Γ, the Li/Na-*s* orbitals belong to *a*1*g* representations, while the host unoccupied *p* state belongs to the *t*1*u* representation. Since there are no common representations between the Li/Na-*s* orbitals and the host-*p* orbital, the *s*-*p* coupling is forbidden, *i.e.*, *Vs*-*p* = 0. As Li/Na atom jumps to the transition site, the symmetry along Path 1 (*Oh*→*D2h*→*Oh*) and Path 2 (*Oh*→*Td*→*Oh*) reduces to *D*2*h* and *Td*, respectively. It is noted that in *D*2*h* symmetry the Li/NaF6 octahedron with Li/Na at the shared edge is distorted, in which a couple of opposite Li/Na-F bond lengths contracted and the square composed by the other four F atoms on the horizontal plane transformed into rectangle. According to the crystal field splitting, the *p* orbitals split into three singlet *b*1*u*, *b*2*u* and *b*3*u* state, and the *s* orbital is in a singlet *au* state. In spite of the reduced symmetry, there is also no *s-p* coupling in this structure (Table S10). Besides, when Li/Na-ions stay at tetrahedral transition sites, the symmetry reduces to *Td*, in which Li/Na-*s* orbitals belong to *a*1 representations, and the host *p* orbital belongs to the *t*2 representation. As a result, there is also no *s*-*p* coupling in this scenario (Table S10). Because in all of the initial/final states as well as the transition states along Path 1 and Path 2, no *s*-*p* coupling could be found, we may expect that the strain energy introduced by Li+/Na+ migration determine the energy landscape along the migration path.


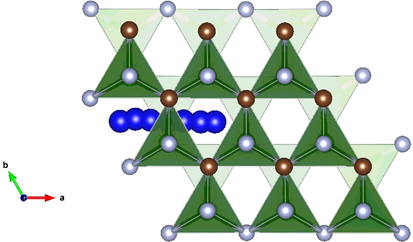

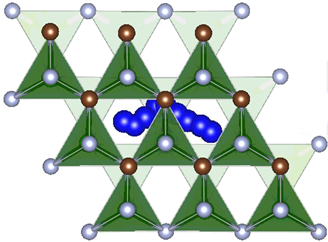

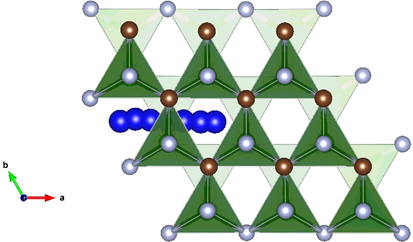

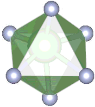

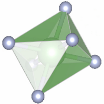

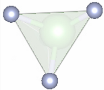


***Oh***

***D2h***

***Td***

**(a) (b)**

Figure S15. Two different pathways, (a) Path 1 (*Oh*→*D2h*→*Oh*) and (b) Path 2 (*Oh*→*Td*→*Oh*) for Li/Na-ions migration in both BCF2 and B2C2F2 systems. The white balls represent F, the green balls represent Li/Na, and the diffusion path of Li/Na atom is highlighted in blue.




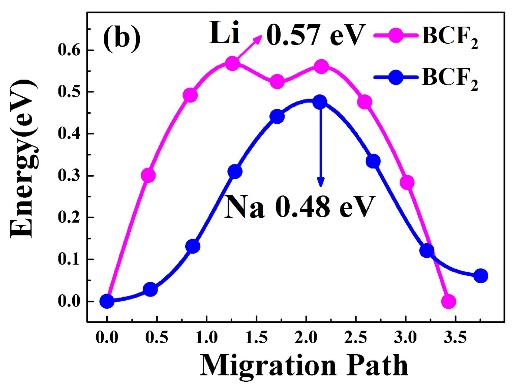




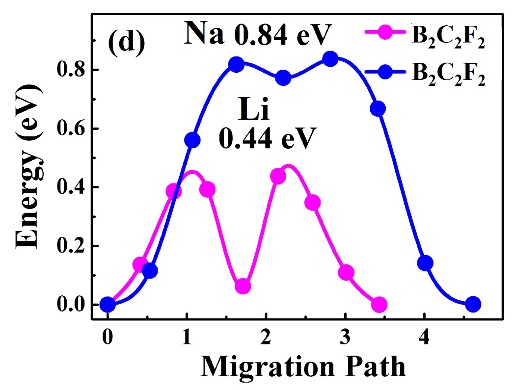


Figure S16. The Li/Na migration energy profiles along Path 1 (*Oh*→*D2h*→*Oh*) by NEB calculations in Li/Na-B-C-F cathodes. (a) and (b) are for Li/NaBCF2 cathodes under discharged and charged states, respectively; (c) and (d) are for Li/NaB2C2F2 cathodes under discharged and charged states, respectively.

Table S9. The Li/Na-ions migration energy barriers (in eV) through two different pathways, Path 1 (*Oh*→*D2h*→*Oh*) and Path 2 (*Oh*→*Td*→*Oh*) in the Li/Na-BCF cathodes.

|  | Li-migration (in eV) | | Na-migration (in eV) | |
| --- | --- | --- | --- | --- |
|  | Path 1  (*Oh*→*D2h*→*Oh*) | Path 2  (*Oh*→*Td*→*Oh*) | Path 1  (*Oh*→*D2h*→*Oh*) | Path 2  (*Oh*→*Td*→*Oh*) |
| Li/NaBCF2  (Discharged States) | 0.67 | 0.70 | 0.70 | 0.73 |
| BCF2  (Charged States) | 0.57 | 0.78 | 0.48 | 1.03 |
| Li/NaB2C2F2  (Discharged States) | 0.70 | 0.81 | 0.88 | 1.36 |
| B2C2F2  (Charged States) | 0.44 | 0.60 | 0.84 | 0.98 |

Table S10. Irreducible representations of Na (Li) *s* and host *p* orbitals in the *Oh*, *D*2*h* and *Td* point groups, respectively. In the *Oh* and *D*2*h* symmetry, there is no *s-p* coupling between the Na (Li) *s* and host *p* orbitals.

| Point group | Na (Li) *s* | Host *p* | *s-p* coupling ? |
| --- | --- | --- | --- |
| *Oh* | *a*1*g* | *t*1*u* | No |
| *D*2*h* | *au* | *b*1*u* ⊕ *b*2*u* ⊕ *b*3*u* | No |
| *Td* | *a*1 | *t*2 | No |

### Section S6: Electrochemical potential platform obtained by the group-subgroup analysis method

Li*x*/Na*x*BCF2 and Li*x*/Na*x*B2C2F2 are composed by the BCF2 and B2C2F2 sheets stacked on each other, which are bound by weak van der Waals forces, respectively, and Li+/Na+ intercalates in the space between these sheets via a sequence of different intercalation reactions. Given that the subtle interplay between these two interactions determines the phase diagram, it is hard to precisely pinpoint the structure for a particular phase in the Li+/Na+ intercalated BCF2/B2C2F2 phase diagram, especially the low ionic content phases, resulting the nebulosity of the full scenario of reaction dynamics. For this reason, A new strategy based on group-subgroup analysis was proposed to carry out a rigorous search over the enormously large configurational phase space with the unit-cell sizes up to 54 Li/Na sites for different intercalation stages of Li*x*/Na*x*-B-C-F systems. The detailed calculation process of our method is as follows:

**S6.1 Algberic group description and subgroups classification of Li/Na-B-C-F**

The Li/Na-B-C-F prototype is trigonal with a space group of *P-3m1* No.164, which can be regarded as a combination of finite point group *D*3*d* and infinite translation group *T*(G) in the perspective of algberic group. Their symmetry operations are shown as follows:

| *D*3d: *P*(G) | (1) 1 | (2) 3+ 0,0, *z* | (3) 3− 0, 0, *z* |
| --- | --- | --- | --- |
|  | (2) 2 *x*,*x*,0 | (5) 2 *x*,0,0 | (6) 2 0, *y*, 0 |
|  | (7) $\bar{1}$ 0,0,0 | (8) $\bar{3}^{+}$ 0, 0, *z*; 0, 0, 0 | (9) $\bar{3}^{-}$ 0, 0, z; 0, 0, 0 |
|  | (10) *m x*, $\bar{x}$, z | (11) *m x*, 2*x*, *z* | (12) m 2*x*, *x*, *z* |
| *T*(G) | =*u*+*v*+*w* | | |
| * , , are basis vector of unit cell, *u*, *v*, *w* are all integer numbers. | | | |

The (*de*)intercalation of Li+/Na+ from host leads to the reduction of group elements (symmetry operations) in either point group *D*3d or translation group *T*(G). This process should be rigorously restrained by subgroup rules. The subgroup *H* of a space group *G* belongs to one of the following three types, which has been reviewed in *International Tables for Crystallography*([32](#_ENREF_32)): (a) translationengleich, or *t*-subgroup, *H* has the same primitive cell as *G* (Figure S17) but the crystal class of *H* is of lower symmetry than that of *G*; or (b) klassengleich, or *k*-subgroup, the crystal class of *H* and *G* is the same (Figure S17) but *H* has lost translational symmetry operations, and thus the primitive cell of *H* is larger than the primitive cell of *G*; or (c) *H* is general, *i.e.*, *H* is neither *translationengleich* nor *klassengleich* and thus when compared with *G*, has lost translational operations as well as crystal class operations. The lattice relationship between subgroup and original one is associated by transformation matrix *P*, $\left( a’, b’, c’ \right)= \left( a, b, c \right)P = \left( a, b, c \right) \left( \begin{matrix} P11 & P12 & P13 \\ P21 & P22 & P23 \\ P31 & P32 & P33 \end{matrix} \right)$.

**1) *t*-subgroups.**

A subgroup *H* < *G* of a space group *G* is called t-subgroup if *T*(G) is retained, i.e. *T*(H) = *T*(G), but the order(number of symmetry operations) of the point group *P*(G)is reduced. t-subgroups are characterized by *t*-index, which indicates number of operations in *P*(G) divided by that in *P*(H). Maximal *t*-subgroups of group *P-3m1* No.164 have been showed as follows:


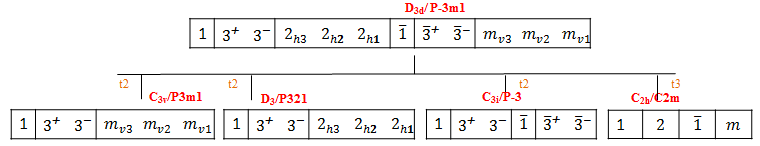


Figure S17. illustration of the maximal *t*-subgroups ofspace group *P-3m1*.

**2) *k*-subgroups**

A subgroup *H* < *G* of a space group *G* is called a *k*-subgroup if the set *T*(G) of all translations of *G* is reduced to *T*(H) < *T*(G), but all point group *P*(G) is retained. *k*-subgroups are characterized by *k*-index, which also indicates the multiplication factor relating the volume of the primitive cell of the subgroup with respect to the primitive cell of the original prototype structure.([33](#_ENREF_33)) *k*-subgroups of Li/Na-B-C-F are further divided in two classes:

•Enlarged unit cell, non-isomorphic.

| [2] c=2c | P$\bar{3}$c1 (165) (2; 7; 4+(0, 0, 1)) a, b, 2c  P$\bar{3}$c1 (165) (2; 4; 7+(0, 0, 1)) a, b, 2c 0, 0, 1/2 |
| --- | --- |
| [3] a=3a,  b=3b | H$\bar{3}$m1 (162, P$\bar{3}$1m) (2; 4; 7) a-b, a+2b, c  H$\bar{3}$m1 (162, P$\bar{3}$1m) ((2; 4)+(1, -1, 0); 7+(2, 0, 0)) a-b, a+2b, c 1, 0, 0  H$\bar{3}$m1 (162, P$\bar{3}$1m) (4; 2+(2, 1, 0); 7+(2, 2, 0)) a-b, a+2b, c 1, 1, 0 |

•Enlarged unit cell, isomorphic.

| [p] c=pc  *P$\bar{3}$m1* (164) | (2; (4; 7)+(0,0,2u))  p > 2; 0 ≤ u < p a, b, pc; 0, 0, u  p conjugate subgroups for the prime p |
| --- | --- |
| [p2]a=pa, b=pb  *P$\bar{3}$m1* (164) | (2+(u+v,-u+2v,0); 4+(u-v,-u+v,0); 7+(2u,2v,0))  p > 1; p ≠ 3; 0 ≤ u < p; 0 ≤ v < p pa, pb, c; u, v, 0  p2 conjugate subgroups for the prime p |

**3) general-subgroups**

Most of the subgroups are general types with elements losing both in point group *P*(G) and translation group *T*(G) during extraction. Therefore, they are characterized by index, a product of *t*-index and *k*-index. The deviation of general subgroups can be finished by integrating *t*-subgroups and *k*-subgroups.

**S6.2 Wyckoff splitting for each pair *G* > *H*.**

Consider group-subgroup related space groups *G*>*H*, atoms which are symmetrically equivalent under *G* (sharing the same Wyckoff position, whose multiplicity indicates how many atoms are equivalent), may become non-equivalent under *H*, and/or their site symmetries may be reduced. This allows to split the high symmetry Wyckoff position (1a) of active ions, corresponding to prototype high-symmetry group of the host (*P-3m1* No.164) into sets of Wyckoff positions referred to the lower symmetry subgroup structure. Each subset can then be occupied independently, thus, the resulting structures are subgroup configurations, where active ions occupies a fraction of the sites. Theoretical aspects of the relations of the Wyckoff positions for a group-subgroup pair *G*>*H* have been demonstrated by Wondratschek([32](#_ENREF_32)), and the program WYCKSPLIT([34](#_ENREF_34)) provides the way handling splitting. An example of Wyckoff splitting in a *t*2*k*3 subgroup is illustrated as follows:

| 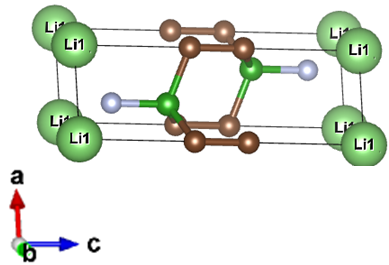 | 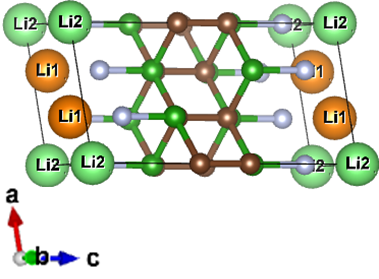 | 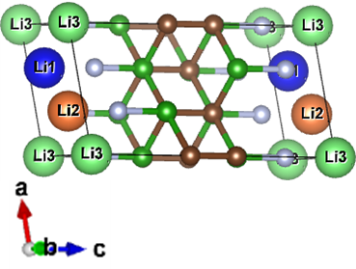 |
| --- | --- | --- |
| space group: *P-3m1* (No.164)  lattice: a,b,c  Li sites: 1a | space group: *P-31m* (No.162)  lattice: a-b, a+2b,c  index: *k*3 of No.164  Li sites: 1a, 2b | space group: *P312* (No.149)  lattice: a-b, a+2b,c  index: *t*2 of No.162  Li sites: 1a, 1a, 1a |

Figure S18. An example of general subgroup obtained from *t*-subgroup and *k*-subgroup. The Wyckoff splitting during group-subgroup projection in the case of projecting NO.164 to No.149 with index 6 was illustrated.

**S6.3 Configurations screening**

We also note that the Ewald electrostatic energy provided by Pymatgen is an essential tool to restraint ab-initio calculations to a relatively small number of configurations due to its validity and accuracy in judging similar structure energies. Ewald summation, named after Paul Peter Ewald is a method for computing long-range interactions (*e.g.*, electrostatic interactions) in periodic systems, which is now commonly used for calculating long-range interactions in computational chemistry.([35](#_ENREF_35)) The method implicitly assumes that the system under study is infinitely periodic. One repeating unit of this hypothetical periodic system is called a unit cell. One such cell is chosen as the "central cell" for reference and the remaining cells are called images. The electrostatic interactions between the particles in periodic system can be represented as $U=\frac{1}{2}\sum_{\overrightarrow{n}} \sum_{u,v=1}^{N} \frac{q_{u}q_{v}}{\left|\overrightarrow{r}_{uv}+\overrightarrow{n}\right|}$, where *ruv* is the distance between particle *u* and *v*, and *n* goes to infinity image cells. It can be seen that the Ewald energy is invariant to the symmetry independent configurations, while it should not be changed for structures that are symmetrically related. Besides, the long-range interaction energy is the sum of interaction energies between the charges of a central unit cell and all the charges of the lattice. The formula is as follows:

*U*Ewald = *U*real + *U*reciprocal + *U*correction

where (Eq. S3)

(Eq. S4)

*U*correction is a constant depending on the parameter $\alpha$. The converged results should not depend on the choice of $\alpha$. This method for configurations screening is validated by Li*x*B2C2F2 (0 < *x* < 1). About 500 sample configurations are picked out from every subgroup of varied *k*-values, then, a comparison between ewald and DFT has been made and the results is show in Figure S19-S20. It is shown that we are able to screen out most of the symmetric similar configurations through ewald energy to save the computational resources. 16220 and 79050 Li*x*/Na*x*BCF2 and Li*x*/Na*x*B2C2F2 configurations in different concentrations are obtained, respectively, which contains all the trigonal subgroups. After screening and eliminating the symmetric similar structures by using the Ewald electrostatic energy method, a total of 2347 and 1337 configurations were calculated to explore the Li*x*/Na*x*BCF2 and Li*x*/Na*x*B2C2F2 ground state hull in our study.


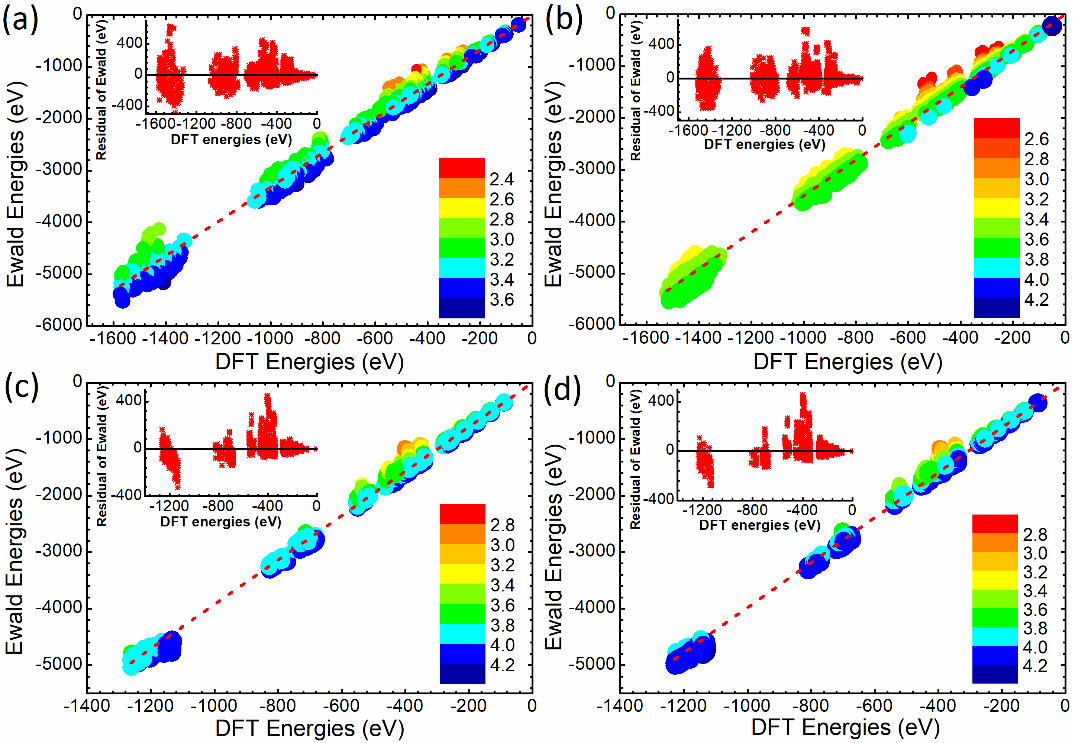


Figure S19. The calculated DFT versus Ewald energies of (a) LiBCF2, (b) NaBCF2, (c) LiB2C2F2 and (d) NaB2C2F2, respectively. The inserts show the linear fitting residual of DFT and Ewald energies, implying that it makes no sense to compare the trend between DFT and Ewald energies.


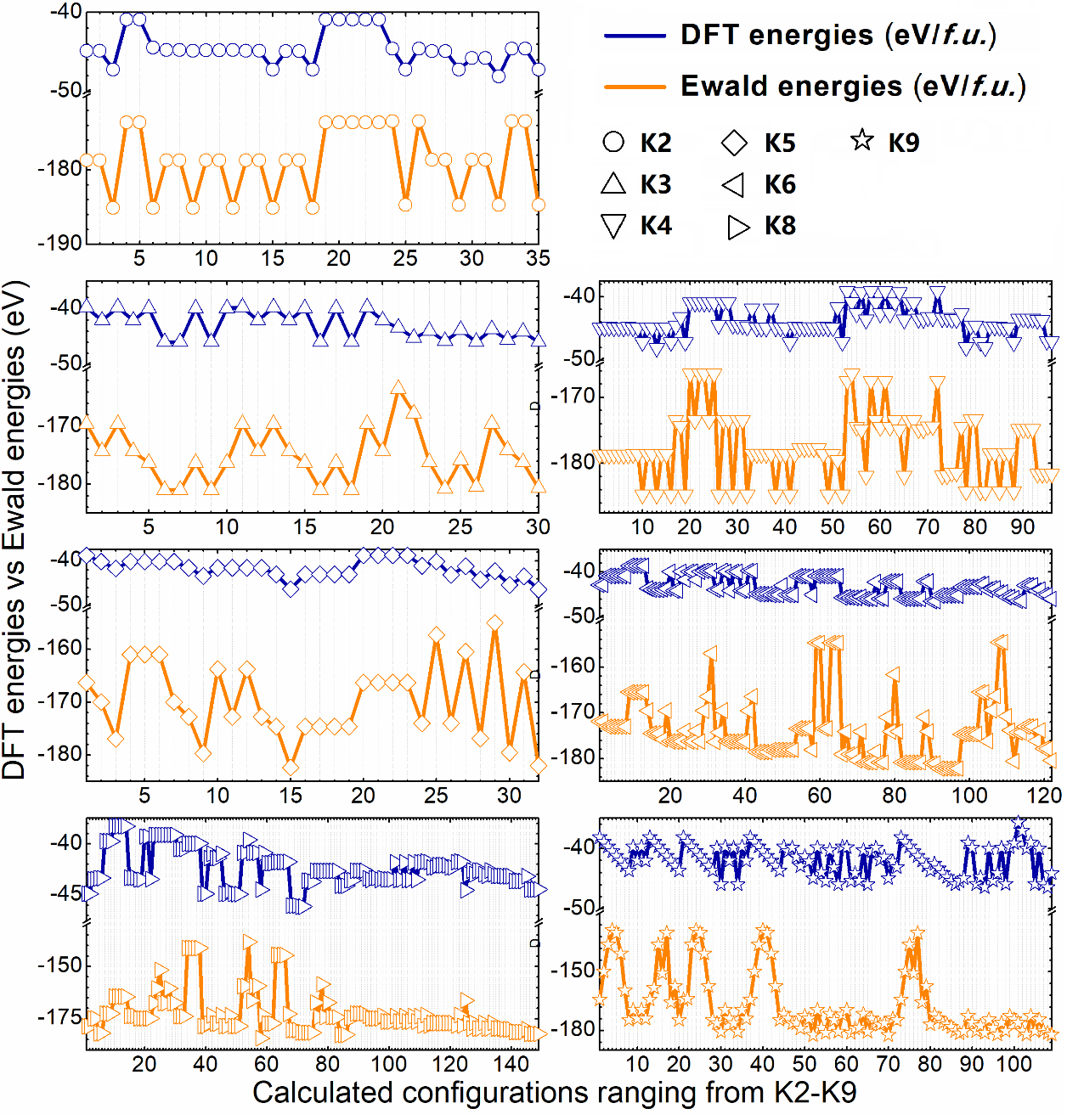


Figure S20. Determining the symmetric similar configurations of Li*x*B2C2F2 (0 < *x* < 1) by DFT and Ewald calculated energies. The *K* values range from 2 to 9. Configurations with the same DFT should have the same Ewald energy. Thus, we are able to screen out most of the symmetric similar configurations through the calculated Ewald energies.

#### S6.4 Formation energy calculations

The formation energy ($\Delta_{f}E_{x}$) of M (M= Li+ or Na+) in B-C-F systems (BCF2 and B2C2F2) are calculated to find the most stable structure for each concentration:

$\Delta_{f}E_{x}=E\left( M_{x}BCF_{2}/M_{x}B_{2}C_{2}F_{2} \right)-[xE\left( MBCF_{2}/MB_{2}C_{2}F_{2} \right)+\left( 1-x \right)E\left( BCF_{2}/B_{2}C_{2}F_{2} \right)]$ (Eq. S5)

where $E(M_{x}BCF_{2}/M_{x}B_{2}C_{2}F_{2})$ is the total energy of the configuration per $M_{x}BCF_{2}$ or $M_{x}B_{2}C_{2}F_{2}$*f.u.*, $E\left( M_{x}BCF_{2}/M_{x}B_{2}C_{2}F_{2} \right)$ and $E\left( BCF_{2}/B_{2}C_{2}F_{2} \right)$ are the energies of $M_{x}BCF_{2}/M_{x}B_{2}C_{2}F_{2}$ and $BCF_{2}/B_{2}C_{2}F_{2}$, respectively. Considering the following electrochemical reaction,

$$M_{x_{1}}BCF_{2}/M_{x_{1}}B_{2}C_{2}F_{2}+\left( x_{2}-x_{1} \right)M^{n+}+n(x_{2}-x_{1})e^{-}\to M_{x_{2}}BCF_{2}/M_{x_{2}}B_{2}C_{2}F_{2}$$

The average intercalation electrochemical potential (Vavg)([36](#_ENREF_36)) of $M_{x}BCF_{2}$ and $M_{x}B_{2}C_{2}F_{2}$ can be determined by,

$V_{avg}=-\frac{E\left( M_{x_{2}}BCF_{2}/M_{x_{2}}B_{2}C_{2}F_{2} \right)-E\left( M_{x_{1}}BCF_{2}/M_{x_{1}}B_{2}C_{2}F_{2} \right)-(x_{2}-x_{1})E_{M}}{n(x_{2}-x_{1})e^{-}}$ (Eq. S6)

where refers to the number of electron transferred. $E\left( M_{x}BCF_{2}/M_{x}B_{2}C_{2}F_{2} \right)$ and $E_{M}$ indicate the total energy of M (=Li+ or Na+) insertion into the structures and bulk M respectively. From Figure 4 and Figure S22, the higher electrochemical potentials were found for BCF2 → Li0.125BCF2 (Stage II, 4.57 V), BCF2 → Na0.062BCF2 (Stage II, 4.61 V), Na0.062BCF2 → Na0.125BCF2 (Stage II, 4.26 V), B2C2F2 → Li0.167B2C2F2 (Stage II, 4.73 V) and B2C2F2 → Na0.111B2C2F2 (Stage III, 5.43 V), which suggest a preference for forming high stage structures during the initial Li+/Na+ intercalation process. Further intercalation of Li+/Na+ into a single layer would lead to a strong repulsive interaction between ions, which is larger than the energy required to expand the interlayer separation. As a result, the Stage I structures with Li+/Na+ intercalation in all layers would be dominant in the following process (Figure S23). The presented framework comprising of statistical group-subgroup analysis and DFT calculations accurately predicts the phase diagram and intercalation potentials for electrochemical Li/Na intercalation in natural BCF2 and B2C2F2. Besides, we identify the following stable phases in the Li+/Na+ intercalation diagram, *viz.*, Na0.111B2C2F2 which is a Stage III compound, Li0.125BCF2, Na0.062BCF2, Na0.125BCF2, Li0.167B2C2F2 which are Stage II compounds, and Li*x*BCF2 (*x* > 0.125), Na*x*BCF2 (*x* > 0.125), Li*x*B2C2F2 (*x* > 0.167), Na*x*B2C2F2 (*x* > 0.111) which are Stage I compounds, and Stage IV could not be found in all (Li/Na)*x*BCF2 and (Li/Na)*x*B2C2F2 intercalation processes (Figure 4).

**Na Concentration**

**Na Concentration**


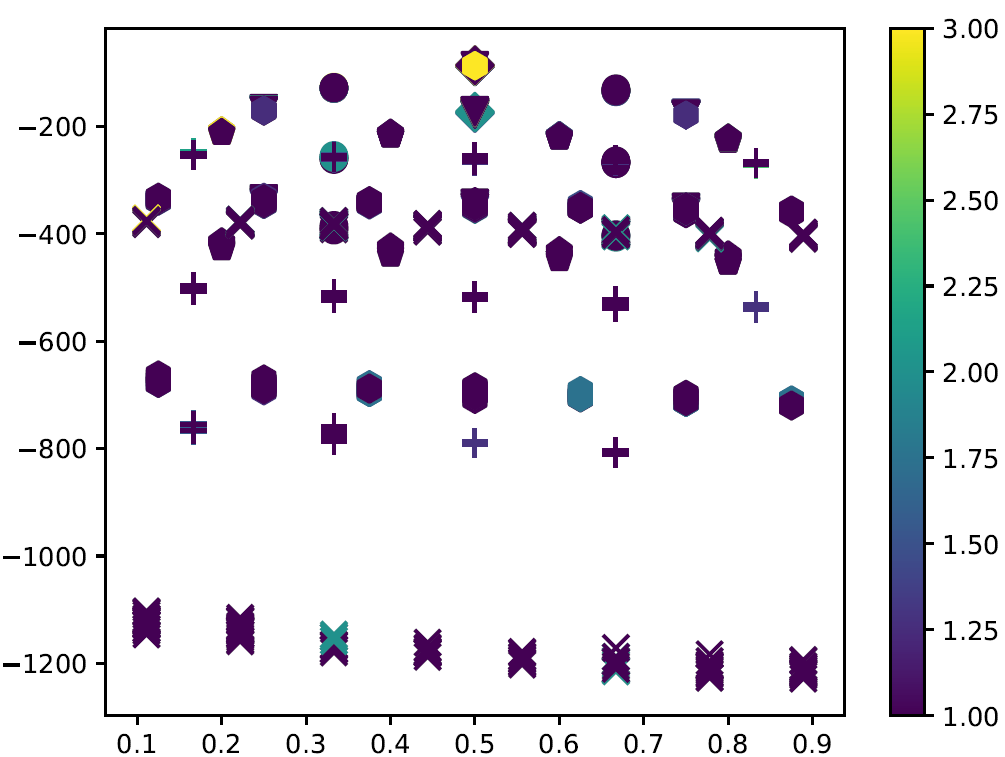

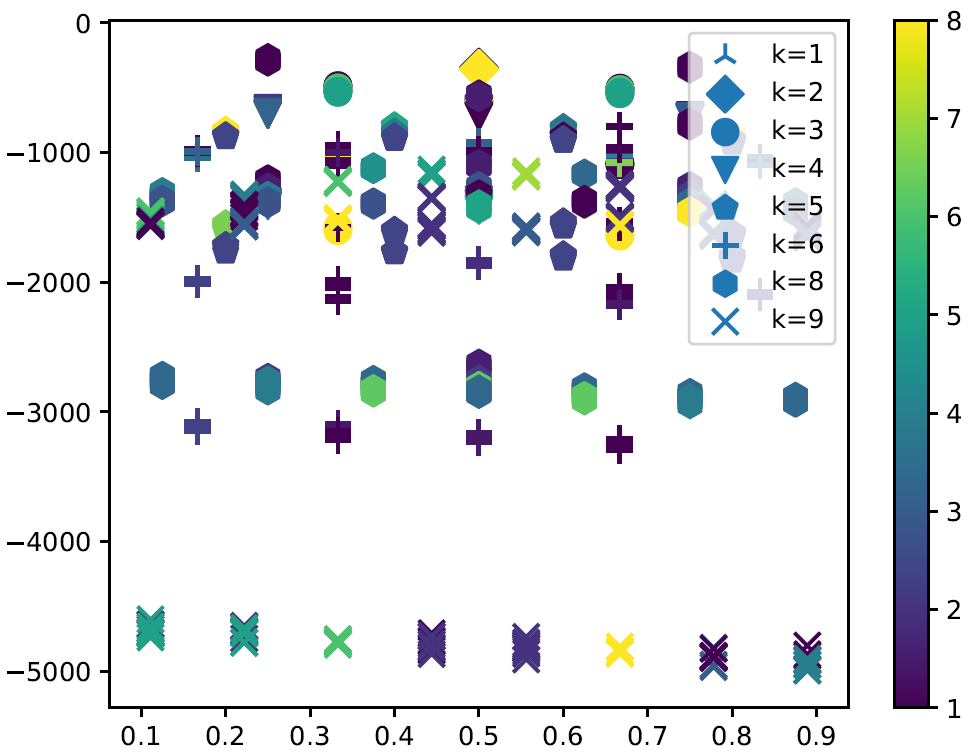


**DFT Energy (eV)**

**Ewald Energy (eV) (eV)**


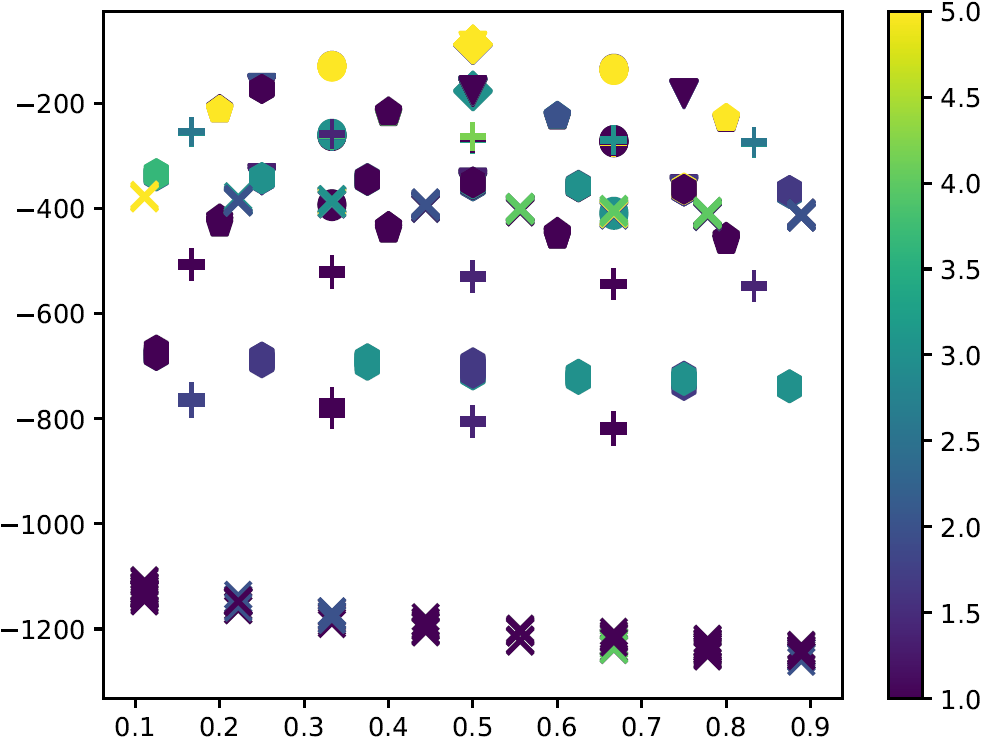

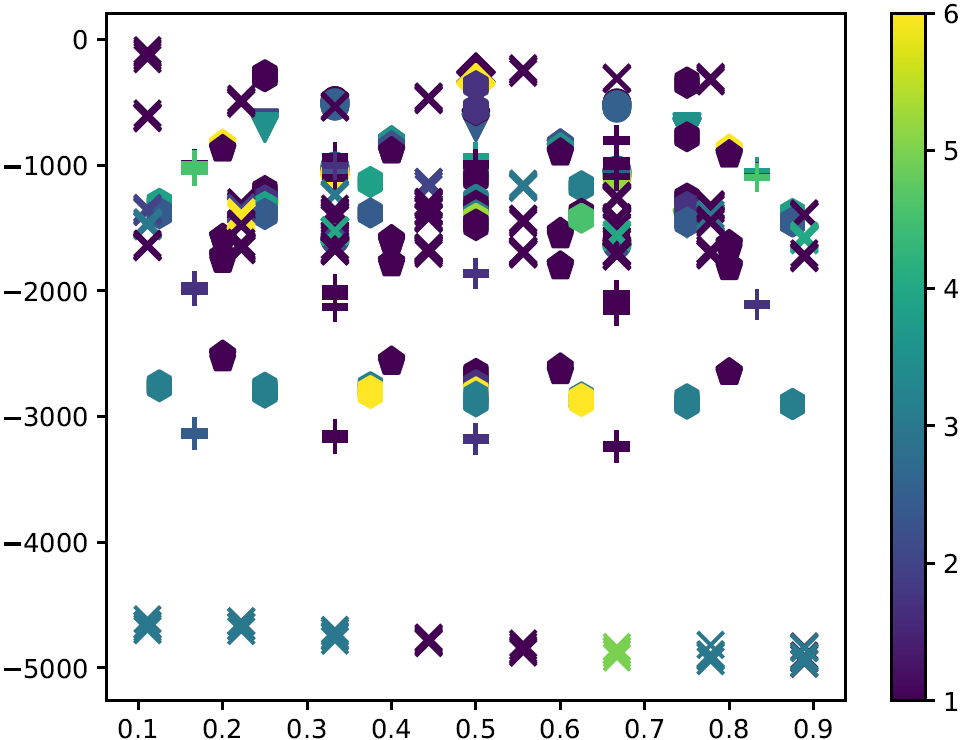


**Ewald Energy (eV) (eV)**

**DFT Energy (eV)**

**Li Concentration**

**Li Concentration**

**~~[使用文档中的独特引言吸引读者的注意力，或者使用此空间强调要点。要在此页面上的任何位置放置此文本框，只需拖动它即可。]Li Concentration~~**


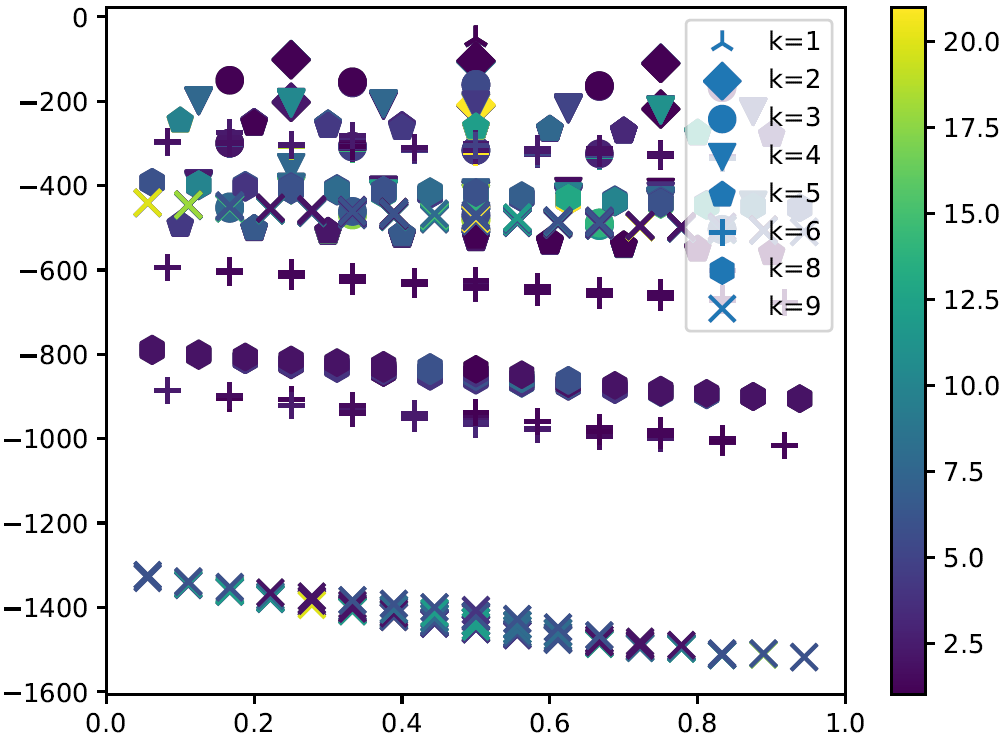

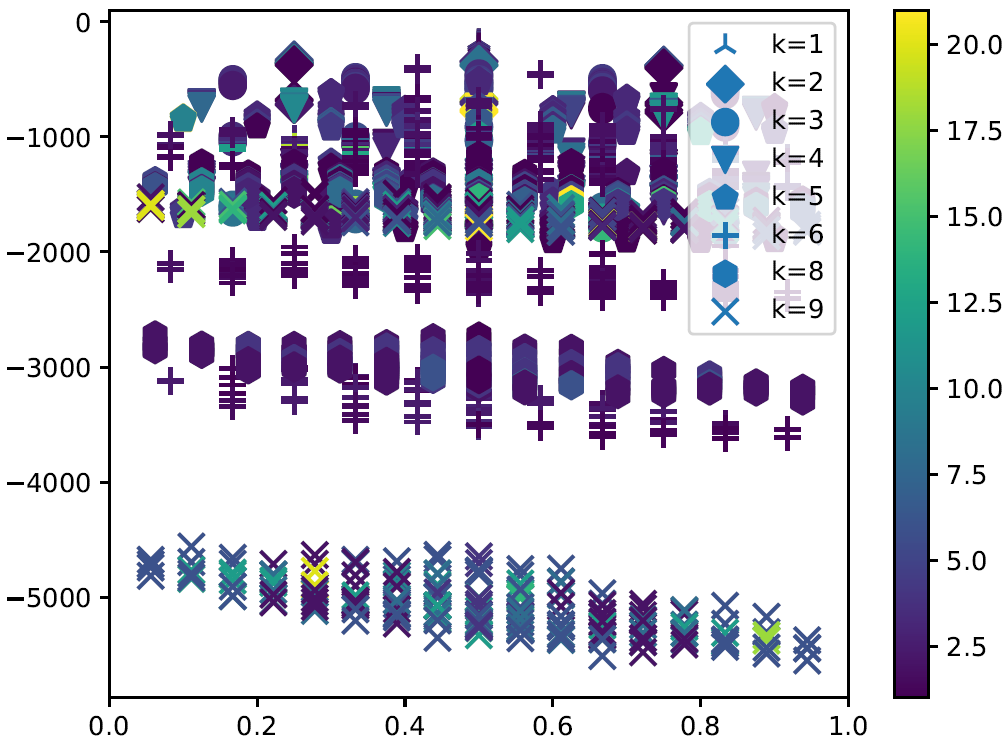


**DFT Energy (eV)**

**Ewald Energy (eV) (eV)**

**Na Concentration**

**Na Concentration**


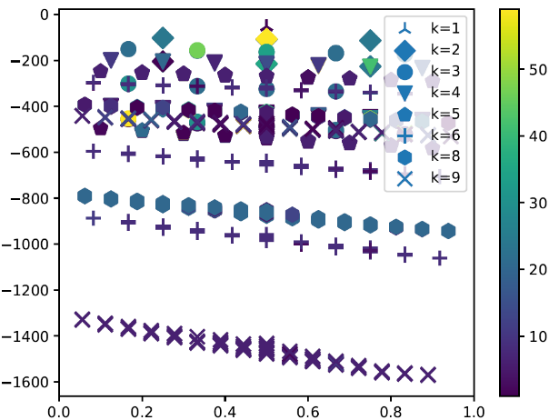

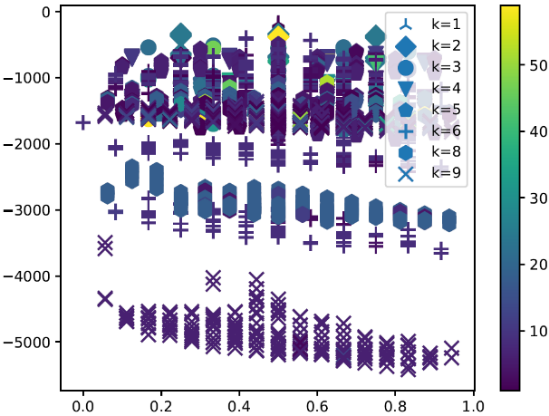


**DFT Energy (eV)**

**Ewald Energy (eV) (eV)**

**Li Concentration**

**Li Concentration**

(a)

(b)

(c)

(d)

Figure S21. The calculated DFT (left) and Ewald (right) energies of (a) LiBCF2, (a) NaBCF2, (b) LiB2C2F2 and (c) NaB2C2F2 as the function of Li/Na-intercalation concentrations, respectively. The K values range from 1 to 9.


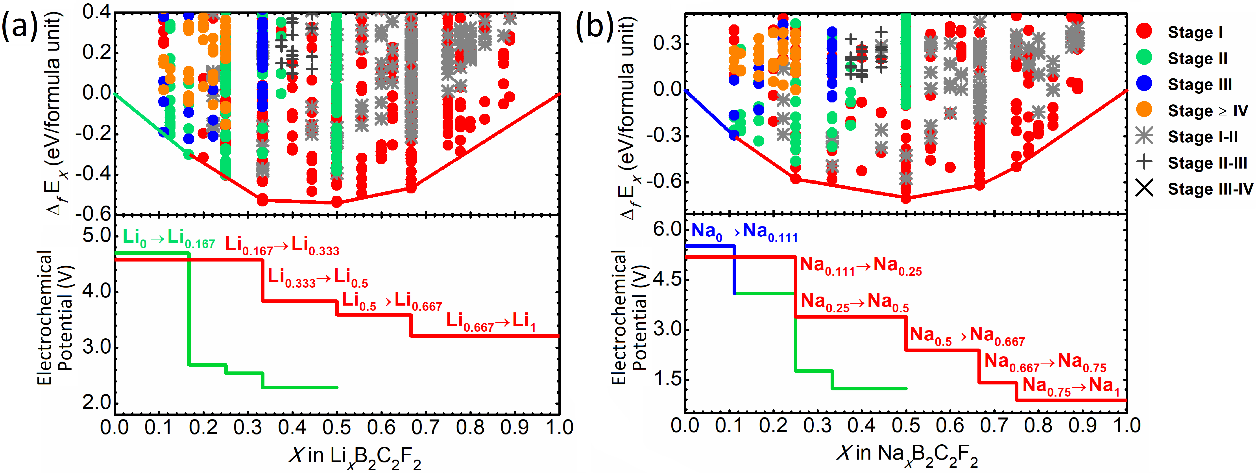


Figure S22. The formation energies (Δ*f*E*x*) per formula unit and the electrochemical potential of (a) Li*x*B2C2F2 and (b) Na*x*B2C2F2 are shown as a function of Li+/Na+ concentration, respectively. The red, green and blue solid lines indicate the constructed convex hull of Stage I, Stage II and Stage III phases, respectively.


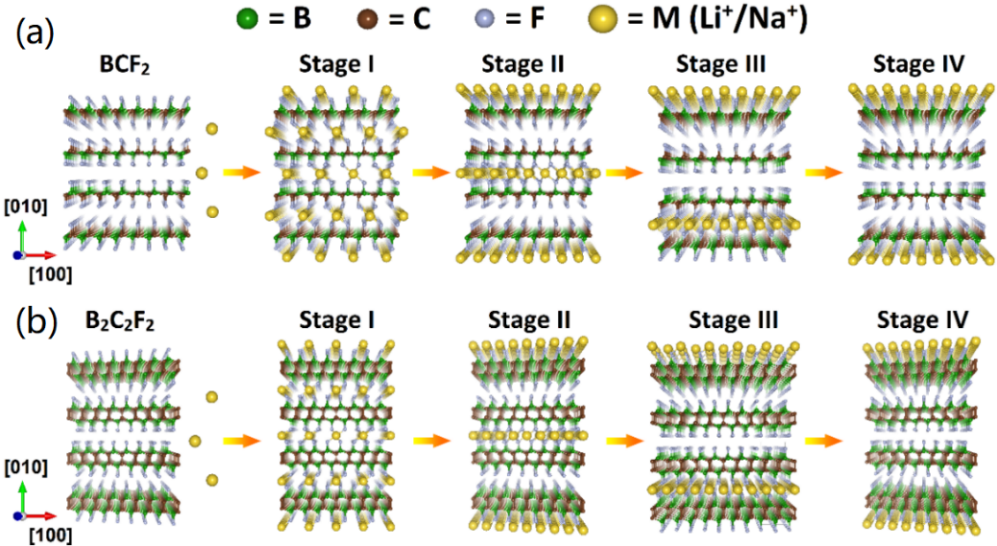


Figure S23. Schematic illustration of different (Stage I-IV) arrangements of Li+/Na+ storage in (a) BCF2 and (b) B2C2F2, respectively.

Table S11. The space group, lattice parameters (Å) and atomic positions of Li*x*BCF2 (0 < *x* < 1) obtained by the group-subgroup analysis method.

| Li0.125BCF2 | Li0.333BCF2 | Li0.5BCF2 | |
| --- | --- | --- | --- |
| C2/m (12)  a, b, c; α, β, γ  9.73, 5.23, 11.73; 90, 90, 90  Site x y z  Li 2a 0.000, 0.000, 0.000  B 4b 0.083, 0.250, 0.235  B 2a 0.333, 0.500, 0.235  B 2a 0.333, 0.000, 0.235  B 4b 0.917, 0.750, -0.235  B 2a 0.167, 0.000, -0.235  B 2a 0.167, 0.500, -0.235  C 4b 0.083, 0.250, 0.737  C 2a 0.333, 0.500, 0.737  C 2a 0.333, 0.000, 0.737  C 4b 0.917, 0.750, -0.737  C 2a 0.167, 0.000, -0.737  C 2a 0.167, 0.500, -0.737  F 4b 0.083, 0.250, 0.109  F 2a 0.333, 0.500, 0.109  F 2a 0.333, 0.000, 0.109  F 4b 0.917, 0.750, -0.109  F 2a 0.167, 0.000, -0.109  F 2a 0.167, 0.500, -0.109  F 4b 0.083, 0.250, 0.392  F 2a 0.333, 0.500, 0.392  F 2a 0.333, 0.000, 0.392  F 4b 0.917, 0.750, -0.392  F 2a 0.167, 0.000, -0.392  F 2a 0.167, 0.500, -0.392 | P2/m (10)  a, b, c; α, β, γ  4.73, 8.15, 11.81, 90, 90, 90  Site x y z  Li 1a 0.000, 0.000, 0.000  Li 1e 0.500, 0.500, 0.000  Li 2k 0.000, 0.333, 0.500  B 2m 0.667, 0.000, 0.235  B 4o 0.667, 0.333, 0.235  B 4o 0.167, 0.167, 0.235  B 2n 0.167, 0.500, 0.235  C 2m 0.667, 0.000, 0.737  C 4o 0.667, 0.333, 0.737  C 4o 0.167, 0.167, 0.737  C 2n 0.167, 0.500, 0.737  F 2m 0.667, 0.000, 0.109  F 4o 0.667, 0.333, 0.109  F 4o 0.167, 0.167, 0.109  F 2n 0.167, 0.500, 0.109  F 2m 0.667, 0.000, 0.392  F 4o 0.667, 0.333, 0.392  F 4o 0.167, 0.167, 0.392  F 2n 0.167, 0.500, 0.392 | | P-31m (162)  a, b, c; α, β, γ  4.74, 4.74, 11.67, 90, 90, 120  Site x y z  Li 18f 0.000, 0.333, 0.000  Li 6c 0.000, 0.000, 0.167  Li 3b 0.000, 0.000, 0.500  B 18f 0.111, 0.222, 0.078  B 18f 0.111, 0.222, 0.412  B 18f 0.111, 0.222, 0.745  C 18f 0.111, 0.222, 0.246  C 18f 0.111, 0.222, 0.579  C 18f 0.111, 0.222, 0.912  F 18f 0.111, 0.222, 0.036  F 18f 0.111, 0.222, 0.370  F 18f 0.111, 0.222, 0.703  F 18f 0.111, 0.222, 0.131  F 18f 0.111, 0.222, 0.464  F 18f 0.111, 0.222, 0.797 |

Table S12. The space group, lattice parameters (Å) and atomic positions of Na*x*BCF2 (0 < *x* < 1) obtained by the group-subgroup analysis method.

| Na0.067BCF2 | Li0.167BCF2 | Li0.5BCF2 | Li0.667BCF2 |
| --- | --- | --- | --- |
| Cm (8)  a, b, c; α, β, γ  9.76, 10.41, 12.18, 90, 90, 90  Site x y z  Na 2a 0.500, 0.500 0.000  B 8j 0.917, 0.875 0.235  B 8j 0.167, 0.250 0.235  B 4i 0.167, 0.500 0.235  B 4i 0.333, 0.500 0.765  B 8j 0.583, 0.875 0.765  C 8j 0.917, 0.875 0.737  C 8j 0.167, 0.250 0.737  C 4i 0.167, 0.500 0.737  C 4i 0.333, 0.500 0.263  C 8j 0.583, 0.875 0.263  F 8j 0.917, 0.875 0.109  F 8j 0.167, 0.250 0.109  F 4i 0.167, 0.500 0.109  F 4i 0.333, 0.500 0.891  F 8j 0.583, 0.875 0.891  F 4i 0.833, 0.000 0.392  F 8j 0.083, 0.625 0.392  F 4i 0.333, 0.000 0.392  F 8j 0.417, 0.625 0.608  F 8j 0.667, 0.750 0.608 | P2/m (10)  a, b, c; α, β, γ  9.73, 5.23, 12.33, 90, 90, 90  Site x y z  Na 2a 0.000, 0.000, 0.000  B 4b 0.083, 0.250, 0.235  B 2a 0.333, 0.500, 0.235  B 2a 0.333, 0.000, 0.235  B 4b 0.917, 0.750, -0.235  B 2a 0.167, 0.000, -0.235  B 2a 0.167, 0.500, -0.235  C 4b 0.083, 0.250, 0.737  C 2a 0.333, 0.500, 0.737  C 2a 0.333, 0.000, 0.737  C 4b 0.917, 0.750, -0.737  C 2a 0.167, 0.000, -0.737  C 2a 0.167, 0.500, -0.737  F 4b 0.083, 0.250, 0.109  F 2a 0.333, 0.500, 0.109  F 2a 0.333, 0.000, 0.109  F 4b 0.917, 0.750, -0.109  F 2a 0.167, 0.000, -0.109  F 2a 0.167, 0.500, -0.109  F 4b 0.084, 0.250, 0.392  F 2a 0.334, 0.500, 0.392  F 2a 0.334, 0.000, 0.392  F 4b 0.917, 0.750, -0.392  F 2a 0.167, 0.000, -0.392  F 2a 0.167, 0.500, -0.392 | C2/m (12)  a, b, c; α, β, γ  4.95, 8.57, 24.90, 90, 90, 90  Site x y z  Na 4g 0.000, 0.333, 0.000  Na 4h 0.000, 0.333, 0.500  Na 4i 0.000, 0.000, 0.250  B 8j 0.167, 0.833, 0.118  B 8j 0.167, 0.833, 0.618  B 4i 0.167, 0.500, 0.118  B 4i 0.167, 0.500, 0.618  C 8j 0.167, 0.833, 0.369  C 8j 0.167, 0.833, 0.869  C 4i 0.167, 0.500, 0.369  C 4i 0.167, 0.500, 0.869  F 8j 0.167, 0.833, 0.054  F 8j 0.167, 0.833, 0.554  F 4i 0.167, 0.500, 0.054  F 4i 0.167, 0.500, 0.554  F 8j 0.167, 0.833, 0.196  F 8j 0.167, 0.833, 0.696  F 4i 0.167, 0.500, 0.196  F 4i 0.167, 0.500, 0.696 | P2/m (10)  a, b, c; α, β, γ  4.95, 8.57, 12.45, 90, 90, 90  Site x, y, z  Na 1a 0.000, 0.000, 0.000  Na 2j 0.500, 0.167, 0.000  Na 1e 0.500, 0.500, 0.000  Na 2k 0.000, 0.333, 0.500  Na 2l 0.500, 0.167, 0.500  B 2m 0.667, 0.000, 0.235  B 4o 0.667, 0.333, 0.235  B 4o 0.167, 0.167, 0.235  B 2n 0.167, 0.500, 0.235  C 2m 0.667, 0.000, 0.737  C 4o 0.667, 0.333, 0.737  C 4o 0.167, 0.167, 0.737  C 2n 0.167, 0.500, 0.737  F 2m 0.667, 0.000, 0.109  F 4o 0.667, 0.333, 0.109  F 4o 0.167, 0.167, 0.109  F 2n 0.167, 0.500, 0.109  F 2m 0.667, 0.000, 0.392  F 4o 0.667, 0.333, 0.392  F 4o 0.167, 0.167, 0.392  F 2n 0.167, 0.500, 0.392 |

Table S13. The space group, lattice parameters (Å) and atomic positions of Li*x*B2C2F2 (0 < *x* < 1) obtained by the group-subgroup analysis method.

| Li0.167B2C2F2 | Li0.333B2C2F2 | Li0.667B2C2F2 | Li0.5B2C2F2 |
| --- | --- | --- | --- |
| P2/m (10)  a, b, c; α, β, γ  4.68, 2.7, 15.65; 90, 90, 90  Site x y z  Li 1a 0.000, 0.000, -1.000  C 2n 0.500, 0.500, -0.198  C 2n 0.500, 0.500, -0.865  C 2n 0.500, 0.500, -0.531  C 2m 0.000, 0.000, -0.198  C 2m 0.000, 0.000, -0.865  C 2m 0.000, 0.000, -0.531  B 2n 0.167, 0.500, -0.220  B 2n 0.167, 0.500, -0.887  B 2n 0.167, 0.500, -0.554  B 2m 0.667, 0.000, -0.220  B 2m 0.667, 0.000, -0.887  B 2m 0.667, 0.000, -0.554  F 2n 0.167, 0.500, -0.047  F 2n 0.167, 0.500, -0.714  F 2n 0.167, 0.500, -0.380  F 2m 0.667, 0.000, -0.047  F 2m 0.667, 0.000, -0.714  F 2m 0.667, 0.000, -0.380 | P-31c (163)  a, b, c; α, β, γ  4.62, 4.62, 16.14; 90, 90, 120  Site x y z  Li 1a 0.000, 0.000, -0.250  Li 1b 0.333, 0.667, -0.750  C 1a 0.000, 0.000, -0.047  C 1a 0.000, 0.000, -0.547  C 1b 0.333, 0.667, -0.047  C 1b 0.333, 0.667, -0.547  C 1c 0.667, 0.333, -0.047  C 1c 0.667, 0.333, -0.547  C 1a 0.000, 0.000, -0.453  C 1a 0.000, 0.000, -0.953  C 1b 0.333, 0.667, -0.453  C 1b 0.333, 0.667, -0.953  C 1c 0.667, 0.333, -0.453  C 1c 0.667, 0.333, -0.953  B 3d 0.000, 0.333, -0.081  B 3d 0.000, 0.333, -0.581  B 3d 0.000, 0.667, -0.419  B 3d 0.000, 0.667, -0.919  F 3d 0.000, 0.337, 0.179  F 3d 0.000, 0.337, -0.321  F 3d 0.000, 0.667, -0.679  F 3d 0.000, 0.667, -1.179 | P-31c (163)  a, b, c; α, β, γ  4.67, 4.67, 15.86; 90, 90, 120  Site x y z  Li 2a 0.000, 0.000, -0.250  Li 2d 0.333, 0.667, -0.250  Li 2c 0.333, 0.667, -0.750  C 4e 0.000, 0.000, -0.047  C 4f 0.333, 0.667, -0.047  C 4f 0.333, 0.667, -0.547  B 12 0.000, -0.333, -0.081  F 12 0.000, -0.333, 0.179 | P2/m (10)  a, b, c; α, β, γ  8.32, 2.71, 4.54; 90, 107.17, 90  Site x, y, z  Li 4c 0.250, 0.000, 0.000  C 4c 0.047, 0.000, 0.000  C 4c 0.047, 0.250, 0.500  C 4c 0.047, 0.500, 0.000  C 4c 0.047, 0.750, 0.500  B 4c 0.747, 0.000, 0.333  B 4c 0.747, 0.250, 0.833  B 4c 0.747, 0.500, 0.333  B 4c 0.747, 0.750, 0.833  F 4c 0.487, 0.000, 0.333  F 4c 0.487, 0.250, 0.833  F 4c 0.487, 0.500, 0.333  F 4c 0.487, 0.750, 0.833 |

Table S14. The space group, lattice parameters (Å) and atomic positions of Na*x*B2C2F2 (0 < *x* < 1) obtained by the group-subgroup analysis method.

| Na0.111B2C2F2 | Na0.25B2C2F2 | Na0.5B2C2F2 | Na0.667B2C2F2 | Na0.75B2C2F2 |
| --- | --- | --- | --- | --- |
| C2/m (12)  a, b, c; α, β, γ  4.59, 8.29, 8.42; 90, 90, 90  Site x y z  Li 3a 0.000, 0.000, 0.000  C 6c 0.000, 0.000, 0.045  C 6c 0.000, 0.000, 0.156  C 6c 0.000, 0.000, 0.267  C 6c 0.000, 0.000, 0.378  C 6c 0.000, 0.000, 0.490  C 6c 0.000, 0.000, 0.601  C 6c 0.000, 0.000, 0.712  C 6c 0.000, 0.000, 0.823  C 6c 0.000, 0.000, 0.934  B 18f 0.000, 0.333, 0.038  B 18f 0.000, 0.333, 0.149  B 18f 0.000, 0.333, 0.260  F 18f 0.000, 0.333, 0.095  F 18f 0.000, 0.333, 0.207  F 18f 0.000, 0.333, 0.318 | C2/m (12)  a, b, c; α, β, γ  17.58, 2.71, 4.61; 90, 76, 90  Site x y z  Li 1a 0.000, 0.000, 0.000  C 1a 0.000, 0.000, 0.203  C 1a 0.000, 0.000, 0.703  C 1b 0.500, 0.500, 0.453  C 1b 0.500, 0.500, 0.953  C 1a 0.000, 0.000, -0.203  C 1a 0.000, 0.000, 0.297  C 1b 0.500, 0.500, 0.047  C 1b 0.500, 0.500, 0.547  B 1b 0.167, 0.500, 0.253  B 1b 0.167, 0.500, 0.753  B 1a 0.667, 0.000, 0.503  B 1a 0.667, 0.000, 1.003  B 1b 0.833, 0.500, 0.747  B 1b 0.833, 0.500, 0.247  B 1a 0.333, 0.000, -0.003  B 1a 0.333, 0.000, 0.497  F 1b 0.167, 0.500, 0.513  F 1b 0.167, 0.500, 1.013  F 1a 0.667, 0.000, 0.763  F 1a 0.667, 0.000, 1.263  F 1b 0.833, 0.500, 0.487  F 1b 0.833, 0.500, -0.013  F 1a 0.333, 0.000, -0.263  F 1a 0.333, 0.000, 0.237 | C2/m (12)  a, b, c; α, β, γ  4.67, 5.48, 16.79; 90, 90, 90  Site x y z  Li 2b 0.500, 0.500, 0.000  C 4g 0.000, 0.000, 0.203  C 4g 0.500, 0.500, 0.203  B 4g 0.667, 0.000, 0.169  B 4g 0.167, 0.500, 0.169  F 4g 0.667, 0.000, 0.429  F 4g 0.167, 0.500, 0.429 | P2/m (10)  a, b, c; α, β, γ  9.52, 2.74, 9.19; 90, 62, 90  Site x y z  Li 8f 0.000, 0.333, 0.000  C 8f 0.000, 0.000, 0.203  C 8f 0.000, 0.333, 0.203  C 8f 0.000, 0.333, 0.703  B 8f 0.167, 0.167, 0.169  B 8f 0.167, 0.167, 0.669  B 8f 0.167, 0.500, 0.169  F 8f 0.167, 0.167, 0.429  F 8f 0.167, 0.167, 0.929  F 8f 0.167, 0.500, 0.429 | P-31m (162)  a, b, c; α, β, γ  4.66, 4.66, 24.33; 90,90,120  Site x y z  Li 2a 0.000, 0.000, 0.000  Li 4e 0.500, 0.250, 0.000  C 4e 0.000, 0.000, 0.203  C 4e 0.000, 0.000, 0.703  C 4e 0.500, 0.250, 0.203  C 4e 0.500, 0.750, 0.203  B 4e 0.167, 0.250, 0.169  B 4e 0.167, 0.750, 0.169  B 4e 0.667, 0.500, 0.169  B 4e 0.667, 0.500, 0.669  F 4e 0.167, 0.250, 0.429  F 4e 0.167, 0.750, 0.429  F 4e 0.667, 0.500, 0.429  F 4e 0.667, 0.500, 0.929 |

#### S6.5 Stability of Li*x*(Na*x*)-B-C-F cathodes upon Li(Na)-ions (de)intercalation

We next investigate the mechanism of charge compensation of Li*x*(Na*x*)BCF2 and Li*x*(Na*x*)B2C2F2 (0 ≤ *x* ≤ 1) during cycling as well as its effect on the stability of cathodes for Li(Na)-ion batteries. Whether the formation of different defects in these materials during cycling would occur is demonstrated to be essentially determined by the energy level of their highest occupied electronic states and can be understood by the defect charge transition mechanism.([37](#_ENREF_37)) In our work, the stability of Li*x*(Na*x*)BCF2 and Li*x*(Na*x*)B2C2F2 cathodes upon Li(Na) (*de*)intercalation was estimated by calculating the formation energy of F vacancy (VF) ([38](#_ENREF_38), [39](#_ENREF_39)). The formation energies of VF are determined according to:

ΔH(VF) = E(VF) – E(host) + E(F) + μ(F) (Eq. S7)

where E(VF), E(host), E(F), and μ(F) are total energies of systems with VF, pristine, the elemental energy of F2 and chemical potential of F. The chemical potential range of F is calculated based on the equilibrium growth conditions for both Li(Na)BCF2 and Li(Na)B2C2F2, where

μ(B) + μ(C) + 2μ(F) = ΔH(BCF2) = -10.39 eV; μ(B) < 0; μ(C) < 0; μ(F) < 0

2μ(B) + 2μ(C) + 2μ(F) = ΔH(B2C2F2) = -6.33 eV; μ(B) < 0; μ(C) < 0; μ(F) < 0 (Eq. S8)

The chemical potential range is shown in Figure S24, which shows 0 eV > μ(F) > -5.20 eV for Li(Na)BCF2 and 0 eV > μ(F) > -3.16 eV for Li(Na)B2C2F2­ when considering BCF2 or B2C2F2 as the only secondary phase for Li(Na)BCF2 or Li(Na)B2C2F2. We would emphasize that either in open oxide or fluoride electrodes under the temperature range of 200 to 500 K (much wider comparing with the battery operating condition), most phase equilibria changes are solid-state reactions involving the absorption or loss of oxygen/fluorine, which is also a major contribution to battery safety.([40-43](#_ENREF_40)) Since the reaction entropy is dominated by the gas entropy, the effect of partial pressure and temperature can be considered to be predominantly captured by change in oxygen/fluorine chemical potential([41](#_ENREF_41)):

$\mu_{gas}\left( T, p_{gas} \right)=\mu_{gas}\left( T, p_{0} \right)+kT\frac{p_{gas}}{p_{0}}=E_{gas}+kT-TS_{gas}^{T,p_{0}}+kYln\frac{p_{gas}}{p_{0}}$ (Eq. S9)

where $p_{0}$ is a reference oxygen/fluorine partial pressure, $p_{gas}$ is the oxygen/fluorine gas partial pressure, k is Boltzmann’s constant, $E_{gas}$ is the gas energy, $S_{gas}^{T,p_{0}}$ is the gas entropy. Thus, the chemical potential of fluorine environment (μ(F) = 0 eV for Eq. S7) is generally considered during the battery operation. Some characteristics can be identified. Firstly, a linear relationship between number of deintercalation Li(Na) and ΔH(VF) is found in Figure S25 for all Li18B18C18F36, Na18B18C18F36, Li18B36C36F36 and Na18B36C36F36 cathodes based on the calculations on $3\times3\times1$ supercell. More importantly, it is shown that all the Li*x*(Na*x*)BCF2 and Li*x*(Na*x*)B2C2F2 cathodes remain stable [ΔH(VF) > 0] at any x, including x=0 when all Li(Na) atoms (total 18 in current supercell) are deintercalated


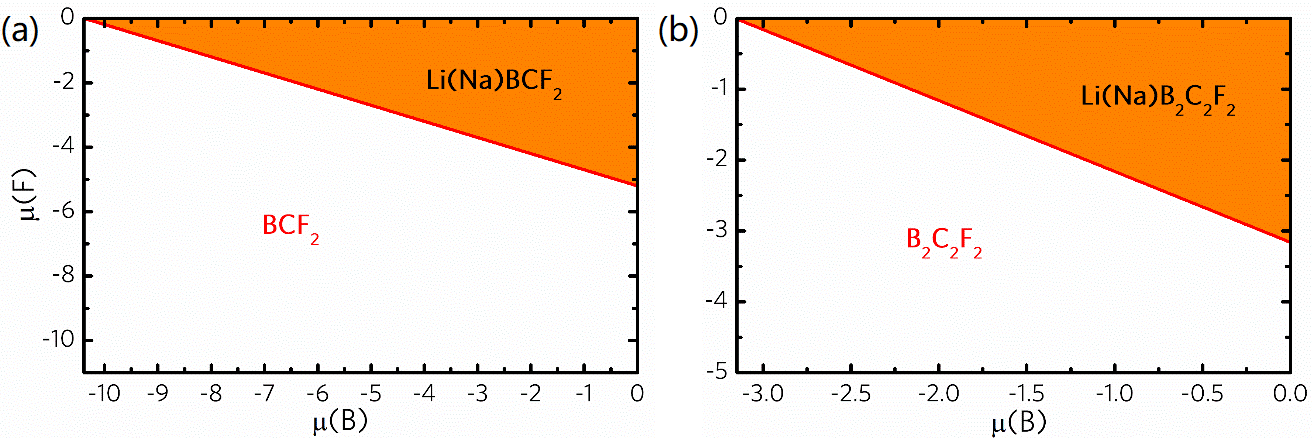


Figure S24. Chemical potential ranges (upper right triangular area) that include the equilibrium growth conditions for (a) Li(Na)BCF2 and (b) Li(Na)B2C2F2, respectively.


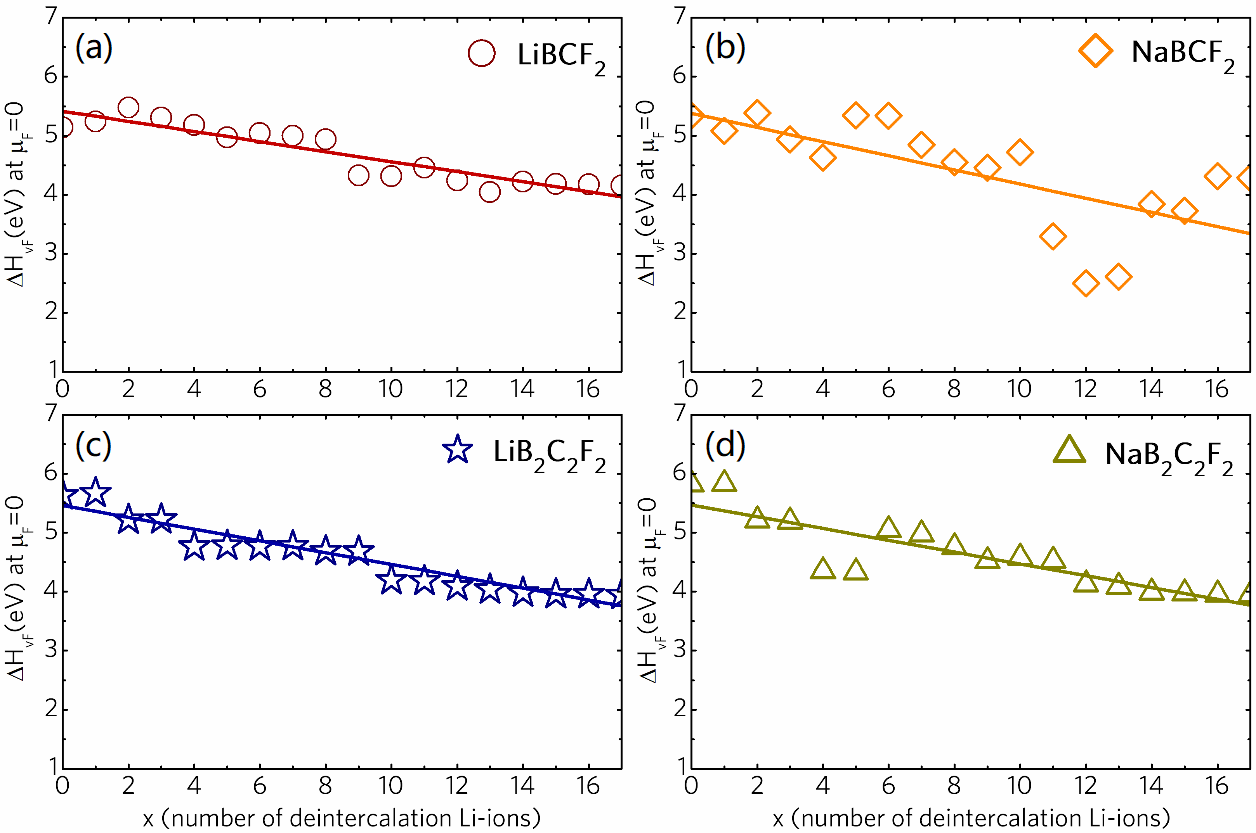


Figure S25. DFT calculated VF formation energy with the increasing number of deintercalation Li(Na)-ions for (a) LiBCF2, (b) NaBCF2, (c) LiB2C2F2 and (d) NaB2C2F2 at μ(F) = 0 eV, respectively.

### References

1. Kresse, G, Furthmüller, J. Efficient iterative schemes for ab initio total-energy calculations using a plane-wave basis set. *Phys Rev B*. 1996; **54**(16): 11169-86.

2. Blöchl, PE. Projector augmented-wave method. *Phys Rev B*. 1994; **50**(24): 17953-79.

3. Perdew, JP, Burke, K, Ernzerhof, M. Generalized gradient approximation made simple. *Phys Rev Lett*. 1996; **77**(18): 3865-8.

4. Monkhorst, HJ, Pack, JD. Special points for Brillouin-zone integrations. *Phys Rev B*. 1976; **13**(12): 5188-92.

5. Grimme, S. Semiempirical GGA-type density functional constructed with a long-range dispersion correction. *J Comput Chem*. 2006; **27**(15): 1787-99.

6. Henkelman, G, Uberuaga, BP, Jónsson, H. A climbing image nudged elastic band method for finding saddle points and minimum energy paths. *J Chem Phys* 2000; **113**(22): 9901-4.

7. Togo, A, Tanaka, I. First principles phonon calculations in materials science. *Scripta Mater*. 2015; **108**: 1-5.

8. Baroni, S, de Gironcoli, S, Dal Corso, A*, et al.* Phonons and related crystal properties from density-functional perturbation theory. *Rev Mod Phys*. 2001; **73**(2): 515-62.

9. Henkelman, G, Arnaldsson, A, Jónsson, H. A fast and robust algorithm for Bader decomposition of charge density. *Comp Mater Sci*. 2006; **36**(3): 354-60.

10. Nosé, S. A unified formulation of the constant temperature molecular dynamics methods. *J Chem Phys*. 1984; **81**(1): 511-9.

11. Guerard, D, Herold, A. Intercalation of lithium into graphite and other carbons. *Carbon*. 1975; **13**(4): 337-45.

12. Xiao, B, Li, YC, Yu, XF*, et al.* Penta-graphene: A promising anode material as the Li/Na-Ion battery with both extremely high theoretical capacity and fast charge/discharge rate. *Acs Appl Mater Inter*. 2016; **8**(51): 35342-52.

13. Thomas, S, Nam, EB, Lee, SU. Atomistic dynamics investigation of the thermomechanical properties and Li diffusion kinetics in psi-Graphene for LIB anode material. *Acs Appl Mater Inter*. 2018; **10**(42): 36240-8.

14. Yu, S, Rao, Y-C, Li, S-F*, et al.* Net W monolayer: A high-performance electrode material for Li-ion batteries. *Appl Phys Lett*. 2018; **112**(5): 053903.

15. Wang, Z, Zhou, XF, Zhang, X*, et al.* Phagraphene: A low-energy graphene allotrope composed of 5-6-7 carbon rings with distorted Dirac cones. *Nano Lett*. 2015; **15**(9): 6182-6.

16. Wang, S, Yang, B, Chen, H*, et al.* Popgraphene: a new 2D planar carbon allotrope composed of 5–8–5 carbon rings for high-performance lithium-ion battery anodes from bottom-up programming. *J Mater Chem A*. 2018; **6**(16): 6815-21.

17. Wang, XQ, Li, HD, Wang, JT. Prediction of a new two-dimensional metallic carbon allotrope. *Phys Chem Chem Phys*. 2013; **15**(6): 2024-30.

18. Ferguson, D, Searles, DJ, Hankel, M. Biphenylene and phagraphene as lithium ion battery anode materials. *Acs Appl Mater Inter*. 2017; **9**(24): 20577-84.

19. Sun, C, Searles, DJ. Lithium storage on graphdiyne predicted by DFT calculations. *J Phys Chem C*. 2012; **116**(50): 26222-6.

20. Liu, J, Wang, S, Sun, Q. All-carbon-based porous topological semimetal for Li-ion battery anode material. *Proc Natl Acad Sci*. 2017; **114**(4): 651-6.

21. Liu, J, Zhao, T, Zhang, S*, et al.* A new metallic carbon allotrope with high stability and potential for lithium ion battery anode material. *Nano Energy*. 2017; **38**: 263-70.

22. Liu, J, Wang, S, Qie, Y*, et al.* High-pressure-assisted design of porous topological semimetal carbon for Li-ion battery anode with high-rate performance. *Phys Rev Mater*. 2018; **2**(2).

23. Liu, J, Li, X, Wang, Q*, et al.* A new 3D Dirac nodal-line semi-metallic graphene monolith for lithium ion battery anode materials. *J Mater Chem A*. 2018; **6**(28): 13816-24.

24. Wang, Y, Lv, J, Zhu, L*, et al.* Crystal structure prediction via particle-swarm optimization. *Phys Rev B*. 2010; **82**(9): 094116.

25. Wang, Y, Lv, J, Zhu, L*, et al.* CALYPSO: A method for crystal structure prediction. *Comput Phys Commun*. 2012; **183**(10): 2063-70.

26. Lv, J, Wang, Y, Zhu, L*, et al.* Predicted novel high-pressure phases of lithium. *Phys Rev Lett*. 2011; **106**(1): 015503.

27. Zhu, L, Wang, H, Wang, Y*, et al.* Substitutional alloy of Bi and Te at high pressure. *Phys Rev Lett*. 2011; **106**(14): 145501.

28. Barber, CB, Dobkin, DP, Dobkin, DP*, et al.* The quickhull algorithm for convex hulls. *ACM Trans Math Softw*. 1996; **22**(4): 469-83.

29. Ong, SP, Wang, L, Kang, B*, et al.* Li-Fe-P-O2 Phase diagram from first principles calculations. *Chem Mater*. 2008; **20**(5): 1798-807.

30. Sanchez, JM, de Fontaine, D. The fee Ising model in the cluster variation approximation. *Phys Rev B*. 1978; **17**(7): 2926-36.

31. Kresse, G, Hafner, J. Ab initio molecular dynamics for liquid metals. *Phys Rev B*. 1993; **47**(1): 558-61.

32. Wonderatschek, H, Müller, U. *International Tables for Crystallography: Volume A1: Symmetry Relations Between Space Groups*: Springer; 2004.

33. Kozinsky, B, Akhade, SA, Hirel, P*, et al.* Effects of sublattice symmetry and frustration on ionic transport in garnet solid electrolytes. *Phys Rev Lett*. 2016; **116**(5): 055901.

34. Aroyo, MI, Perez-Mato, J, Orobengoa, D*, et al.* Crystallography online: Bilbao crystallographic server. *Bulg. Chem Commun*. 2011; **43**(2): 183-97.

35. Ewald, PP. Die berechnung optischer und elektrostatischer gitterpotentiale. *ann Phys-berlin*. 1921; **369**(3): 253-87.

36. Aydinol, MK, Kohan, AF, Ceder, G*, et al.* Ab initio study of lithium intercalation in metal oxides and metal dichalcogenides. *Phys Rev B*. 1997; **56**(3): 1354-65.

37. Zhang, P, Wei, S-H. Origin of charge compensation and its effect on the stability of oxide cathodes for Li-ion batteries: The case of orthosilicates. *Electrochim Acta*. 2018; **270**: 409-16.

38. Yabuuchi, N, Nakayama, M, Takeuchi, M*, et al.* Origin of stabilization and destabilization in solid-state redox reaction of oxide ions for lithium-ion batteries. *Nat Commun*. 2016; **7**(1): 13814.

39. Lv, D, Bai, J, Zhang, P*, et al.* Understanding the high capacity of Li2FeSiO4: in situ XRD/XANES study combined with first-principles calculations. *Chem Mater*. 2013; **25**(10): 2014-20.

40. Lee, J, Papp, JK, Clément, RJ*, et al.* Mitigating oxygen loss to improve the cycling performance of high capacity cation-disordered cathode materials. *Nat Commun*. 2017; **8**(1): 981.

41. Ong, SP, Jain, A, Hautier, G*, et al.* Thermal stabilities of delithiated olivine MPO4 (M=Fe, Mn) cathodes investigated using first principles calculations. *Electrochem Commun*. 2010; **12**(3): 427-30.

42. Larsson, F, Andersson, P, Blomqvist, P*, et al.* Toxic fluoride gas emissions from lithium-ion battery fires. *Sci Rep*. 2017; **7**(1): 10018.

43. Hamwi, A, Guérin, K, Dubois, M. Chapter 17-Fluorine-intercalated graphite for lithium batteries. In: Nakajima, T, Groult, H (eds.). *Fluorinated materials for energy conversion*. Amsterdam: Elsevier Science; 2005. 369-95.
